# Integrated transcriptomic and single-cell RNA sequencing identifies lysosomal ion channel genes as potential biomarkers for Alzheimer’s disease

**DOI:** 10.3389/fgene.2025.1676565

**Published:** 2025-10-08

**Authors:** Xin Wang, Zelin Wu, Shaoli Wei, Xinran Zhao, Juan Lin, Fang Zhao, Xiaolei Liu

**Affiliations:** Third Hospital of Shanxi Medical University, Shanxi Bethune Hospital, Shanxi Academy of Medical Sciences, Tongji Shanxi Hospital, Taiyuan, China

**Keywords:** Alzheimer’s disease, lysosomal ion channels, single cell analysis, nomogram, lysosomal-immune homeostasis

## Abstract

Previous research has highlighted lysosomal ion channel-related genes (LICRGs) as promising therapeutic targets for neurodegenerative diseases. This study aimed to identify and analyze LICRG-associated biomarkers for Alzheimer’s disease (AD), elucidating their underlying biological mechanisms. Three datasets (GSE63061, GSE63060, GSE181279) were analyzed. In GSE63061, intersecting genes were identified by integrating differentially expressed genes (DEGs) from differential expression analysis with key module genes from Weighted Gene Co-expression Network Analysis (WGCNA). Candidate biomarkers were then selected using the MCODE plugin for PPI analysis (top 30 genes), two machine learning approaches, and cross-validation of gene expression profiles in GSE63061 and GSE63060. Single-cell RNA sequencing (scRNA-seq) analysis of GSE181279 identified key biomarkers and cell populations, followed by pseudo-temporal analysis of these cells. Nomogram construction, functional enrichment analysis, immune infiltration assessment, and RT-qPCR analysis were subsequently performed. scRNA-seq analysis revealed that *SRP14*, *EIF3E*, and *COX7C* were prominently expressed across most cell types, particularly in CD4^+^ T cells, which were identified as key cells in AD. Pseudo-temporal analysis indicated that CD4^+^ T cells from control subjects primarily resided in early differentiation stages, whereas those from patients with AD were predominantly found in later stages. The reduced expression of these biomarkers in AD CD4^+^ T cells was consistent with transcriptomic data and further validated by RT-qPCR. A nomogram incorporating these biomarkers demonstrated strong predictive power for AD risk. Functional analysis linked the biomarkers to pathways such as “ribosome” and “oxidative phosphorylation.” Immune infiltration analysis revealed 23 differentially abundant immune cell types, with significant correlations between all three biomarkers and memory CD4^+^ T cells, mesangial cells, and other immune cell types. This study identified *SRP14*, *EIF3E*, and *COX7C* as novel biomarkers, underscoring CD4^+^ T cells as pivotal in AD pathogenesis. These findings offer new mechanistic insights and potential therapeutic strategies for AD.

## 1 Introduction

Alzheimer’s disease (AD), the most common neurodegenerative disorder, is characterized by progressive cognitive decline and neuropathological features, including amyloid-beta (Aβ) plaques and neurofibrillary tangles. By 2050, the global prevalence of AD is projected to surpass 138 million cases, placing an enormous socioeconomic burden on society (Scheltens et al., 2021). Current treatments, such as acetylcholinesterase inhibitors and NMDA antagonists, only alleviate symptoms rather than halt disease progression, highlighting the urgent need to elucidate the molecular mechanisms underlying AD and identify novel therapeutic targets (Lane et al., 2018).

Lysosomal dysfunction has emerged as a central factor in AD pathogenesis. Lysosomes, acidic organelles responsible for cellular waste degradation, regulate Aβ clearance and neuronal homeostasis (Wani et al., 2021). Recent studies have identified lysosomal ion channels, including TRPML1 (Peng et al., 2020), TPCs (Wang et al., 2012), and TMEM175 (Cang et al., 2015), as critical regulators of lysosomal pH, ion balance, and autophagic flux, which are essential for preventing toxic protein aggregation (Riederer et al., 2023). For instance, TRPML1, an endosomal and lysosomal Ca^2+^-releasing channel, accelerates the degradation and clearance of intracellular Aβ by promoting autophagosome-lysosome fusion (via the p62/dynein pathway) and enhancing axonal transport and brain-derived neurotrophic factor (BDNF) signaling. TRPML1 activation amplifies these processes (Zhang et al., 2022; Poon et al., 2011; Kurosawa et al., 2016). In models of HIV infection-associated Aβ accumulation, TRPML1 activation stimulates lysosomal exocytosis and Aβ clearance. ML-SA1 reduced elevated sphingomyelin levels (long-chain/very-long-chain) in the cortex of triple-transgenic gp120/APP/PS1 mice, alleviating TRPML1 inhibition by sphingomyelins and forming a positive feedback loop of “calcium release - sphingomyelin clearance - TRPML1 recovery,” ultimately promoting Aβ clearance (Somogyi et al., 2023; Bae et al., 2014). However, the systematic characterization of lysosomal ion channel-related genes (LICRGs) in AD—especially their cell-type-specific roles and interactions with immune cells—remains insufficiently explored.

Single-cell RNA sequencing (scRNA-seq) offers unprecedented resolution for dissecting cellular heterogeneity in AD (Aldridge and Teichmann, 2020). Recent scRNA-seq studies have uncovered dysregulated microglial subtypes and T-cell infiltration in AD brains, underscoring the role of neuroinflammation in disease progression (Sun et al., 2024). However, the involvement of LICRGs in immune cell dysfunction, such as CD4^+^ T cell differentiation and cytokine signaling, remains poorly understood. Furthermore, many existing studies on LICRGs rely on bulk transcriptomic analyses, which obscure cell-specific expression patterns.

In this study, LICRGs were taken as the focal point, and a comprehensive research framework was adopted: “multi-dataset integration - multi-method key gene screening - single-cell localization of functional carriers - multi-dimensional mechanism elucidation - clinical translation validation.” This approach ultimately identified key genes and cell populations, elucidated the potential mechanisms of AD progression, and provided valuable diagnostic models and therapeutic targets, thereby offering a novel direction for AD mechanism research and clinical intervention.

## 2 Materials and methods

### 2.1 Data extraction

Transcriptomic datasets related to AD, specifically GSE63061 and GSE63060 (Sood et al., 2015), were downloaded from the Gene Expression Omnibus (GEO, https://www.ncbi.nlm.nih.gov/geo/). GSE63061 (GPL10558 platform) included 139 AD and 134 control blood samples, while GSE63060 (GPL6947 platform) comprised 145 AD and 104 control blood samples. scRNA-seq data from GSE181279 (GPL24676 platform) (Xu and Jia, 2021), also obtained from GEO, included immune cell samples from three patients with AD and two healthy controls. Additionally, six LICRGs—TPC1, TPC2, TMEM175, TRPML1, CLN1, and CLC-7—were selected based on prior research (Riederer et al., 2023).

### 2.2 Differential expression analysis

Differentially expressed genes (DEGs) between AD and control samples in GSE63061 were identified using the “limma” package (version 3.56.2) (Ritchie et al., 2015), with thresholds set at |log_2_ fold-change (FC)| ≥ 0.2 and *P* < 0.05. DEGs were visualized through volcano plots (ggplot2, version 3.3.6) (Gustavsson et al., 2022) and heatmaps (heatmap3, version 1.1.9) (Zhao et al., 2014). Genes were ranked based on |log_2_FC| (in descending order), with the volcano plot highlighting the top five most significant up-/downregulated genes, and the heatmap displaying the top ten.

### 2.3 WGCNA

In GSE63061, LICRG scores for samples were calculated using the single-sample Gene Set Enrichment Analysis (ssGSEA) algorithm (Barbie et al., 2009), with LICRGs as the background gene set. Disparities in LICRG scores between the AD and control cohorts were evaluated and found to be statistically significant (P < 0.05). Additionally, Weighted Gene Co-expression Network Analysis (WGCNA, version 1.7.1) (Langfelder and Horvath, 2008) was used to identify key modules. Cluster analysis of all GSE63061 samples excluded anomalies. The optimal soft threshold and mean connectivity were determined to assess the tightness of gene connections and ensure that the constructed co-expression network approximated a scale-free distribution. Co-expression matrices were then constructed with a minimum module size of 50 genes. Gene modules of different colors were generated, and modules significantly correlated with LICRG scores (|Spearman *cor*| > 0.3 and P < 0.05) were identified. The most strongly correlated modules (with the highest positive/negative *cor*) were selected as key modules, and genes within these pivotal modules were considered hub genes.

### 2.4 Biological characterization of intersection genes and candidate genes identification

Consensus genomic elements were derived from the intersection of differentially expressed transcripts and key network components. Gene Ontology (GO) and Kyoto Encyclopedia of Genes and Genomes (KEGG) pathway analyses were performed to explore the functions of the intersecting genes using the “clusterProfiler” package (version 4.8.2) (Yu et al., 2012) (adjusted P < 0.05). To further investigate protein interactions among the intersecting genes, a protein-protein interaction (PPI) network was constructed using the STRING database (https://string-db.org/) with a confidence score threshold of >0.4. The network was visualized using Cytoscape (version 3.9.1) (Shannon et al., 2003). The MCODE plugin was employed for PPI analysis to identify candidate genes for subsequent validation.

### 2.5 Machine learning and gene expression analysis

The Least Absolute Shrinkage and Selection Operator (LASSO) and Boruta algorithms were applied to screen feature genes from the candidate set. LASSO analysis was performed using the “glmnet” package (version 4.1.4) (En et al., 2019), with results confirmed at the minimum lambda value. The Boruta algorithm, utilizing the “Boruta” package (version 8.0.0) (Zhou et al., 2023), was employed to identify key genes. Genes that intersected between LASSO and Boruta were selected as feature genes. Gene expression analysis of these feature genes was then conducted in the GSE63061 and GSE63060 datasets. Genes showing significant differential expression between AD and control groups, and consistent expression trends across both datasets, were designated as candidate biomarkers.

### 2.6 ScRNA-seq analysis

The scRNA-seq data from the GSE181279 dataset were processed using the “Seurat” package (version 4.3.0) (Hao et al., 2023). Low-quality cells were removed based on quality control (QC) criteria, with thresholds set at 200 < nFeature-RNA <2,500, nCount-RNA <8,000, and percent.mt < 15%. Data normalization was performed using the “LogNormalize” function, and the top 2000 highly variable genes (HVGs) were selected using the vst method from the “FindVariableFeatures” function. Principal component analysis (PCA) was then performed on the 2000 HVGs using the “RunPCA” function, and sample integration was conducted using the RPCAIntegration method within the “IntegrateLayers” function. Principal components (PCs) were selected based on an elbow plot. The cells were subsequently clustered using t-distributed stochastic neighbor embedding (t-SNE) with a resolution of 1. Cell annotation was performed using “singleR” (version 2.0.0) (Huang et al., 2021) based on marker genes identified through literature mining (Xu and Jia, 2021). Expression of the proposed biomarkers within annotated cells was evaluated, and genes showing significant expression in these cells were considered biomarkers. The Wilcoxon rank-sum test was used for further analysis. Cells were designated as key cells when all candidate biomarkers demonstrated significant expression differences between AD and control groups (P < 0.05).

### 2.7 Intercellular signaling and pseudotime Trajectory analysis

To investigate interactions between different cell types and gain deeper insight into key cells, cell-to-cell communication analysis was performed using “CellChat” (version 1.6.1) (Luo et al., 2023) in the GSE181279 dataset. Additionally, to explore the differentiation states and trajectories of key cells, pseudo-temporal analysis was conducted using “monocle” (version 2.26.0) (Qiu et al., 2017). Pseudotime series represent abstract biological processes, mapping cellular developmental states onto pseudotime trajectories, calculating gene expression changes over pseudotime, and inferring the developmental state of cells (Yongchun Wang et al., 2024). An expression heatmap of the top 100 genes contributing to cell differentiation was then generated. These 100 genes underwent GO and KEGG pathway enrichment analyses using the “clusterProfiler” package (adj. P < 0.05) to identify pathways significantly altered during cell differentiation.

### 2.8 Development and calibration of a predictive scoring system

A nomogram was constructed based on the identified biomarkers to predict the risk of AD using the “rms” package (version 6.3.0) (Liu et al., 2021). To assess the nomogram’s predictive accuracy, calibration and decision curves were generated. If the P-value of the Hosmer-Lemeshow (HL) test for the calibration curve is greater than 0.05 (indicating good model calibration), the concordance index (C-index) exceeds 0.7 (indicating favorable discriminative ability), and the net benefit from decision curves is non-zero, the model is considered capable of effectively distinguishing patients with AD from healthy controls.

### 2.9 Function analysis of biomarkers

To further explore the biological functions and signaling networks associated with the biomarkers, GSEA was performed. Unlike traditional GO and KEGG enrichment analyses, GSEA identifies changes in pathway activity related to key genes based on “overall trends of gene sets” rather than individual gene function annotations (Aravind Subramanian et al., 2005). This approach was employed to validate and expand the functional enrichment results obtained from GO/KEGG analyses. First, the strength of association (via Spearman correlation) between each biomarker and all other genes in the GSE63061 dataset was determined, with associations ranked from strongest to weakest. The C2: KEGG gene set was downloaded using the “msigdbr” package (version 7.5.1) (Liberzon et al., 2015) as the background set. Subsequently, GSEA was conducted to enrich the ranked genes within the background gene set, with an adjusted P-value threshold set at < 0.05.

### 2.10 The reverse transcription quantitative PCR (RT-qPCR)

A total of 20 blood samples (10 from patients with AD and 10 from healthy controls) were collected from the Shanxi Bethune Hospital. All participants provided informed consent, and the study was approved by the hospital’s ethics committee (approval number: YXLL-2025-151). Total RNA was extracted from the samples using TRIzol reagent (Ambion, United States), following the manufacturer’s protocol. RNA integrity and concentration were assessed using a NanoPhotometer N50. Complementary DNA (cDNA) synthesis was performed using the SureScript First-Strand cDNA Synthesis Kit on a Bio-Rad S1000TM Thermal Cycler. The primer sequences used for the qPCR are listed in S1 Table (Supplemental). Quantitative real-time PCR (qPCR) amplification was carried out with a Bio-Rad CFX Connect Real-Time PCR System. Thermocycling parameters included an initial denaturation step at 95 °C for 1 min, followed by 40 amplification cycles (denaturation: 95 °C for 20 s, primer annealing: 55 °C for 20 s, elongation: 72 °C for 30 s). Gene expression levels were quantified using the 2^−ΔΔCT^ method.

### 2.11 Statistical analysis

Statistical analysis was performed in R (version 4.2.2). Differences between groups were analyzed using the Wilcoxon test (P < 0.05). For comparisons of PCR results between groups, an independent samples t-test was employed (P < 0.05).

## 3 Results

### 3.1 Identification of 134 DEGs and 847 key module genes

**FIGURE 1:**
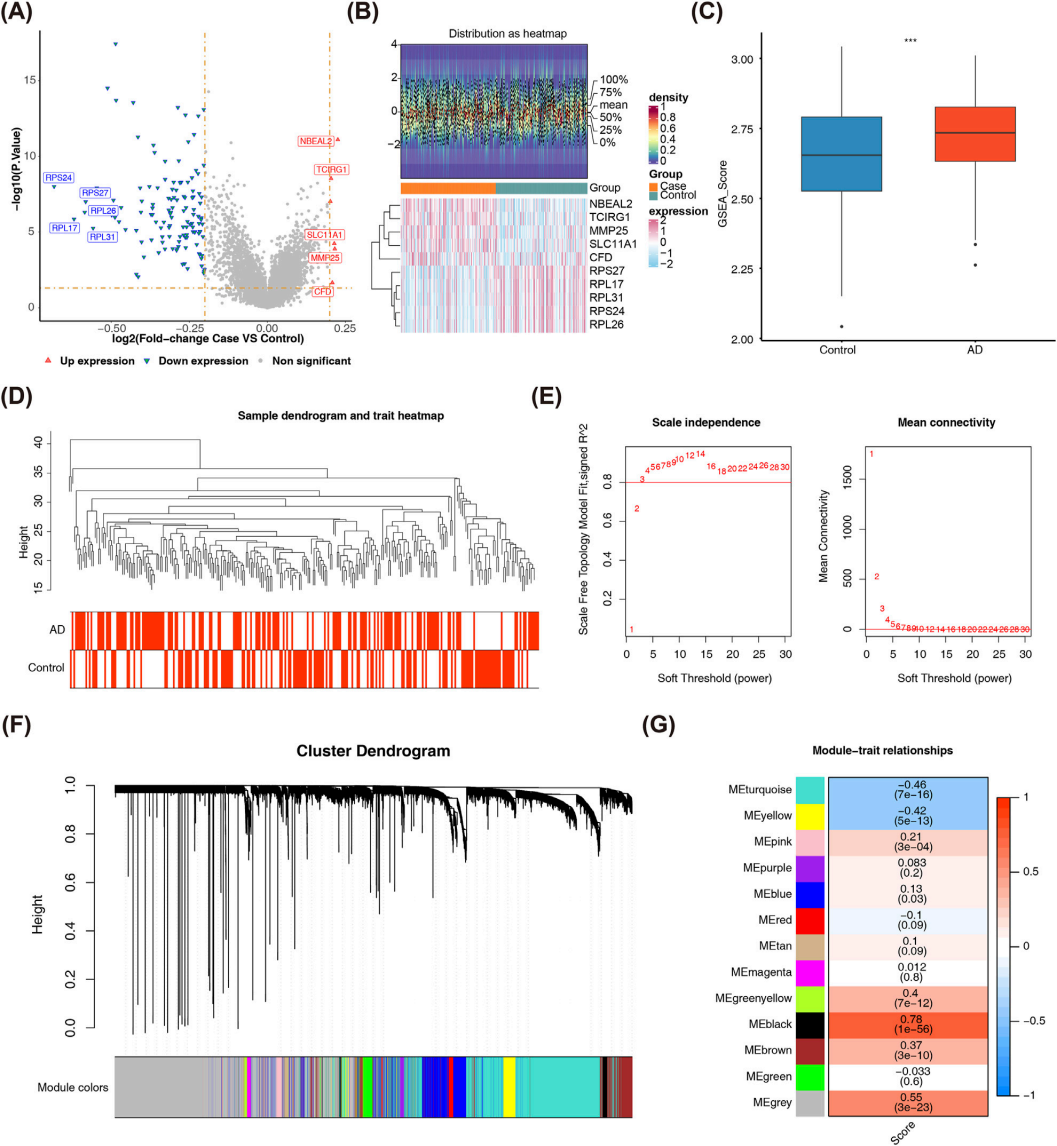
Identification of Key DEGs and Key Modules of Lysosomal Ion Channels. **(A)** The volcano plot illustrates the distribution of differentially expressed genes (DEGs) between patients with 139 AD and 134 control samples in the GSE63061 dataset. Red points denote significantly upregulated genes, while blue points represent significantly downregulated genes. **(B)** The heatmap presents the differential gene expression profiles across samples in the comparison cohort. The upper panel assesses data distribution characteristics, where proximal mean and median values indicate a near-normal distribution. The lower panel employs a color gradient to represent normalized transcript abundance, with red indicating upregulation and blue indicating downregulation. **(C)** Comparison of lysosomal ion channel-related gene (LICRG) scores between patients with AD and control samples reveals a significant difference between the two groups. Statistical significance thresholds: *p < 0.05, **p < 0.01, ***p < 0.001. **(D)** The sample clustering tree shows that all samples were included in subsequent analysis, with no outlier samples detected. The cutreeStatic function was used to identify abnormal samples within the clustering tree. **(E)** The optimal soft-thresholding power determination in WGCNA follows scale-free network topology metrics, with a target fit index approaching 0.8. **(F)** The module clustering tree from WGCNA analysis, with different colors representing distinct gene modules. A total of 13 modules were obtained. **(G)** Inter-modular association profiling highlighted MEblack and MEturquoise as the most strongly correlated WGCNA eigenmodules with LICRG scores.

Differential expression analysis identified 134 DEGs between AD and control groups, including 6 upregulated and 128 downregulated genes (Figures 1A,B). The heatmap confirmed robust data quality and reliable differential analysis, revealing a significant divergence in gene expression patterns between AD and control groups. Specifically, the AD group exhibited a tendency for upregulation (P < 0.05), highlighting coordinated gene expression changes that reflect AD-related biological processes. To identify module genes linked to lysosomal ion channels in the training set, WGCNA was performed. This analysis revealed interconnected gene modules that drive AD pathogenesis, crucial for understanding how individual genes collaborate to contribute to the disease. AD samples exhibited significantly higher LICRG scores than controls (P = 0.00096) (Figure 1C). Analysis of the GSE63061 dataset identified no aberrant samples (samples with values or features that statistically significantly deviate from the majority of observations in the dataset) (Figure 1D), as the cutHeight parameter for the cutreeStatic function in hierarchical clustering was objectively set to 45 based on the dendrogram’s natural branching pattern—ensuring stable cluster separation without over-splitting biologically relevant groups. The optimal soft threshold (β) was determined to be 8, based on the scale-free R^2^ approaching 0.8 and mean connectivity nearing zero, ensuring the network approximated a scale-free distribution (Figure 1E). Modules with high topological overlap were clustered, yielding 13 distinct co-expression groups, each assigned a unique color (Figure 1F). Significant associations were found between LICRG scores and module eigengenes: MEblack demonstrated a strong positive correlation (r = 0.78, P = 1e-56), while MEturquoise exhibited a negative correlation (r = −0.46, P = 7e-16) (Figure 1G). Consequently, the 847 genes in the MEblack and MEturquoise modules were selected as key module genes.

### 3.2 Enrichment of 94 intersection genes in the ribosome pathway

**FIGURE 2:**
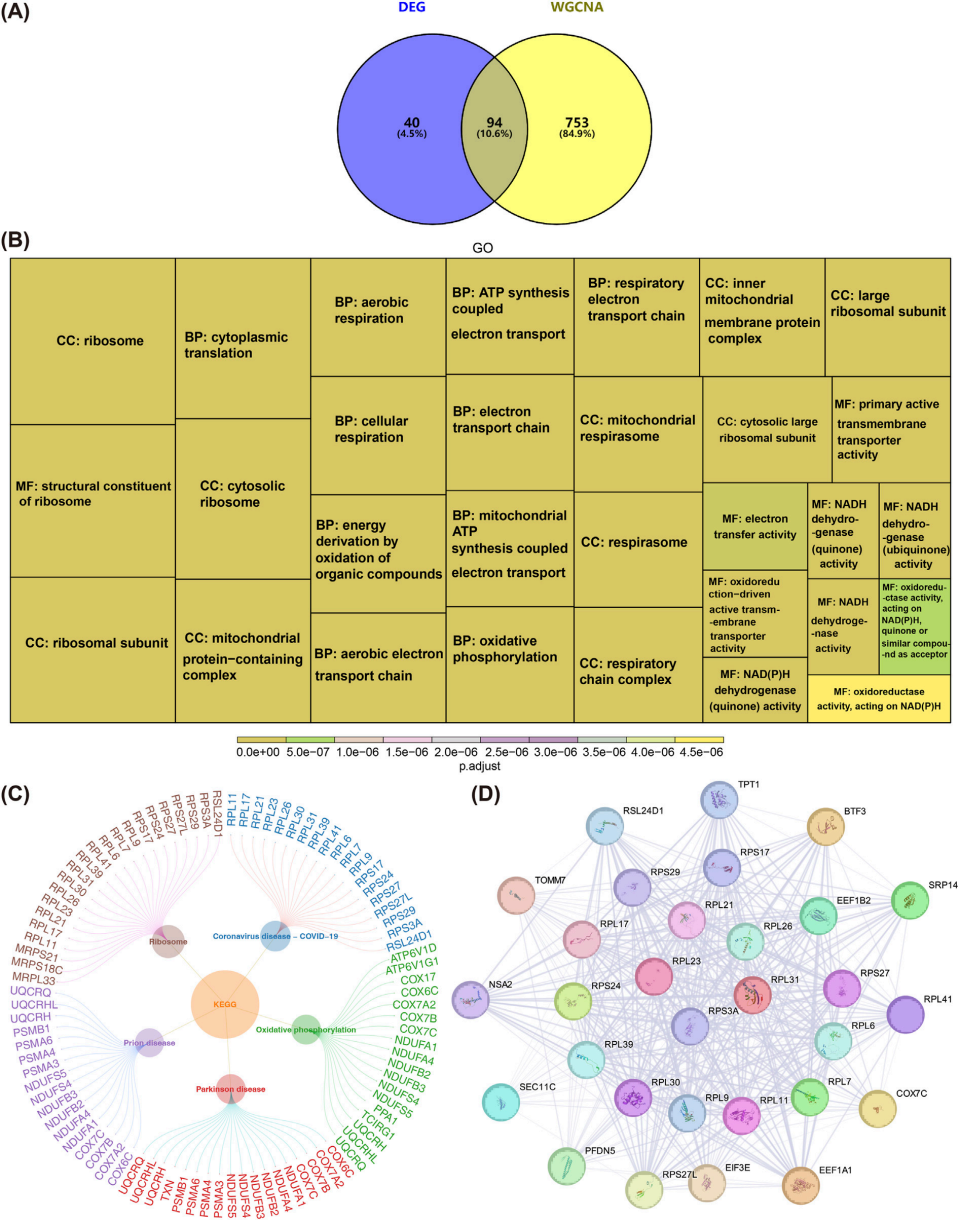
Identification and Functional Enrichment Analysis of Candidate Genes. **(A)** Venn diagram illustrating the intersection of differentially expressed genes (DEGs) and key module genes identified through WGCNA analysis. **(B)** Gene Ontology (GO) enrichment analysis of the 94 candidate genes,and Display the top 30 most significantly enriched functions. **(C)** Kyoto Encyclopedia of Genes and Genomes (KEGG) pathway enrichment analysis of the 94 candidate genes. **(D)** Magnified view of the top 30 genes in the PPI network, constructed using the MCODE plugin. This subnetwork includes 30 nodes and 387 edges, highlighting protein-level interactions among these genes. Red nodes indicate genes associated with core biomarkers.

Venn diagrams were employed to identify DEGs associated with lysosomal ion channels in AD, aiming to filter out “consistently dysregulated genes” that likely drive AD pathogenesis. By overlapping the 134 DEGs with the 847 key module genes, a total of 94 intersecting genes were identified (Figure 2A). Functional enrichment analysis revealed that these 94 genes were significantly enriched in 303 GO terms and 23 KEGG pathways (adj. P < 0.05). Notable GO terms included “ribosome,” “cytoplasmic translation,” and “structural constituent of ribosome,” among others (Figure 2B). The top five KEGG pathways significantly enriched with the intersecting genes were “coronavirus disease-COVID-19,” “oxidative phosphorylation,” “Parkinson’s disease,” “prion disease,” and “ribosome” (Figure 2C). In the context of neurodegenerative diseases, dysfunction in this gene set may lead to increased synthesis of misfolded proteins or ribosomal dysfunction, thereby exacerbating pathological damage (Brilkova et al., 2022). A PPI network was constructed using the 94 intersecting genes, excluding outlier genes, resulting in a network of 90 nodes and 826 edges (Supplementary Figure S1). For example, RPL21 and RPL17 showed strong interactions with other genes. Using the MCODE plugin, a subnetwork comprising the top 30 genes was generated, revealing protein-level interactions among these genes. This subnetwork contained 30 nodes and 387 edges, with genes such as RPS3A and RPL31 exhibiting close interactions (Figure 2D). These 30 genes were selected as candidate targets for further analysis.

### 3.3 Identification of 7 candidate biomarkers

**FIGURE 3:**
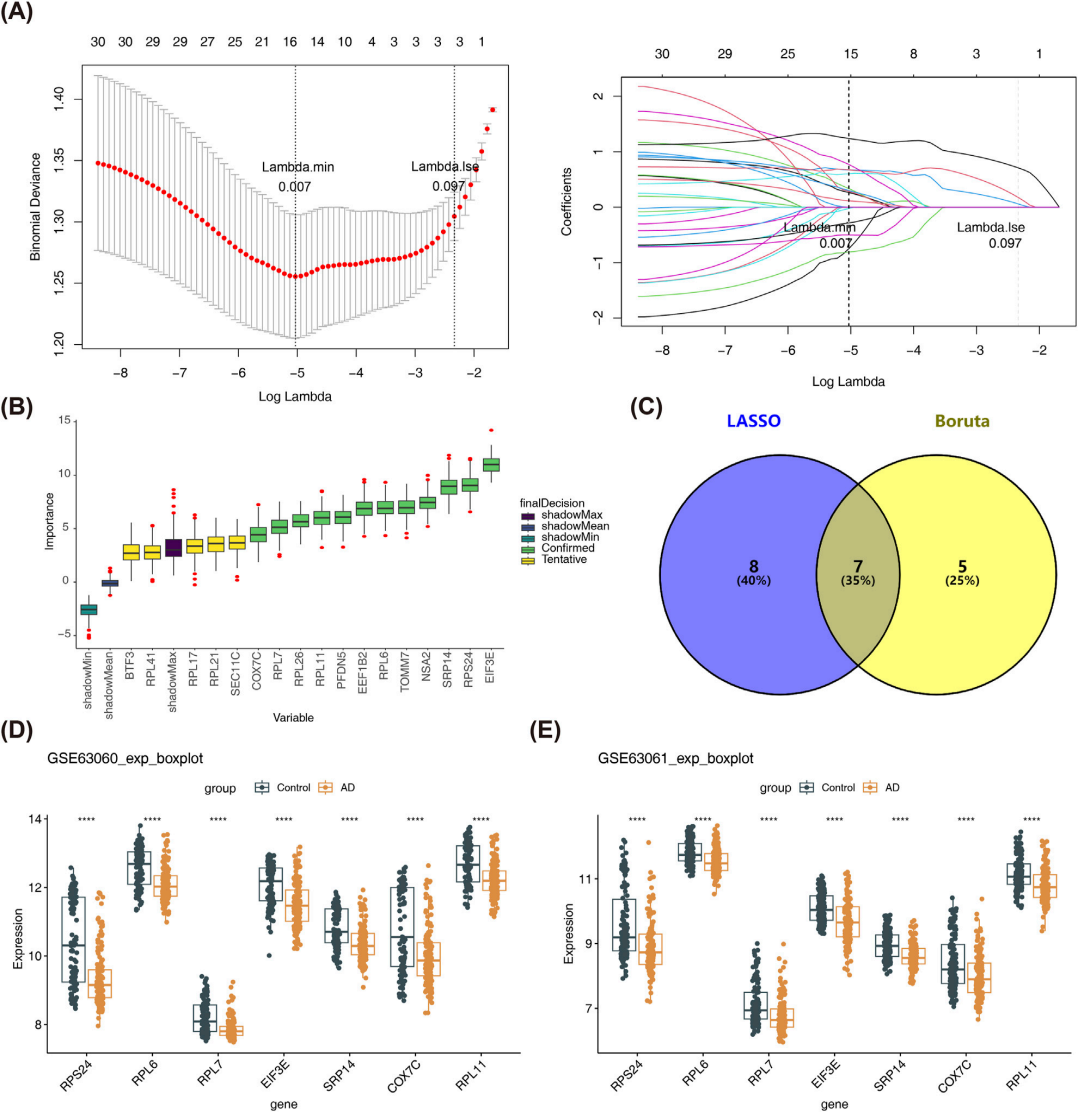
Identification of Candidate Biomarkers. **(A)** LASSO regression analysis was performed, where the lambda (λ) value determines the selection of feature genes. The red vertical line corresponds to the lambda.min value, and the green vertical line represents the lambda value. Genes obtained at the minimum λ value usually correspond to the optimal results. **(B)** The boxplot illustrates the importance scores of the genes, with green boxes indicating confirmed important genes, yellow boxes representing uncertain genes, and the purple box indicating the maximum importance score of the shadow features. **(C)** The Venn diagram depicts the intersection of feature genes identified by LASSO and Boruta analyses. A total of 7 feature genes were identified, including SRP14, RPL11, RPL6, EIF3E, COX7C, RPL7, and RPS24. **(D,E)** The boxplots show the expression levels of the 7 feature genes in the GSE63061 (139 AD and 134 control samples) and GSE63060 (145 AD and 104 control samples) datasets. These genes were significantly downregulated in AD samples compared to control samples in both datasets. Statistical significance thresholds: *p < 0.05, **p < 0.01, ***p < 0.001.

Data mining methods, including LASSO and Boruta, combined with expression level validation, were used to effectively identify candidate biomarkers. The LASSO method (lambda min = 0.007) selected 15 genes from the 30 candidate genes (Figure 3A), while the Boruta method identified 12 genes (Figure 3B). Overlapping the results from both methods led to the identification of 7 feature genes (*SRP14*, *RPL11*, *RPL6*, *EIF3E*, *COX7C*, *RPL7*, and *RPS24*) (Figure 3C). Comparisons between AD and control samples from the GSE63061 and GSE63060 datasets revealed significantly decreased expression of all seven feature genes (*P* < 0.05), designating them as candidate biomarkers for AD (Figures 3D,E).

### 3.4 Biomarker validation and key cell identification

**FIGURE 4:**
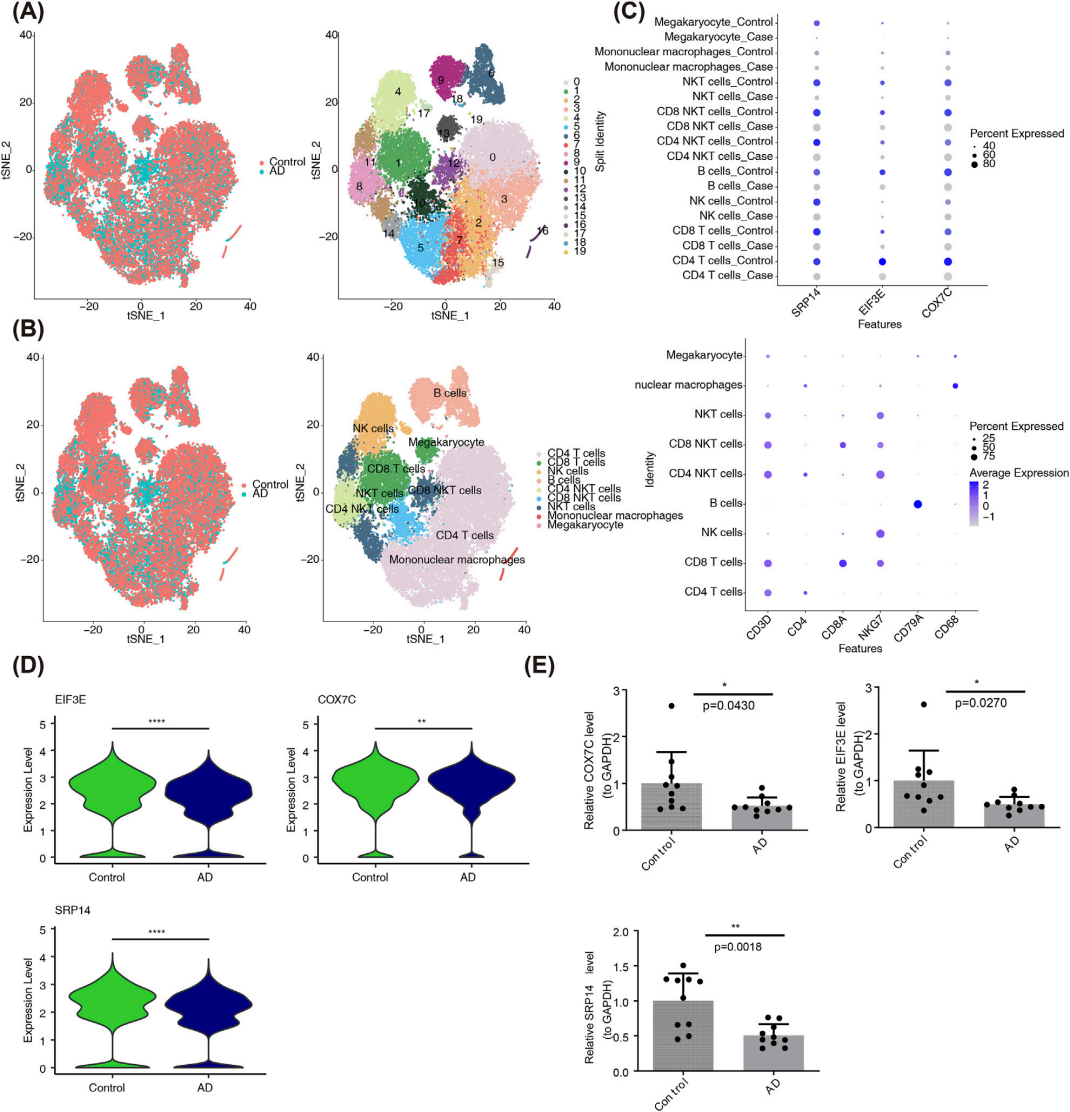
Identification of Key Genes and Cell Types. **(A)** Distribution of cells following dimensionality reduction clustering (Left), and t-SNE clustering results showing distinct cell clusters within the integrated single-cell dataset (Right). Color denotes predefined groups (Left) and identified cell clusters (Right). **(B)** Cell type annotation based on marker genes. The plot displays the annotated cell types, including CD4 T cells, CD8 T cells, NK cells, B cells, CD4 NKT cells, CD8 NKT cells, NKT cells, mononuclear macrophages, and megakaryocytes. **(C)** The heatmap shows the expression levels of candidate biomarkers across different cell types, with significant expression observed in CD4 T cells. The size of the dots represents the expression proportion of the gene in the respective cell cluster, and the color indicates the expression level, where darker colors represent higher expression levels and lighter colors correspond to lower expression levels. **(D)** The boxplot displays the expression levels of SRP14, EIF3E, and COX7C in CD4 T cells from 3 AD and 2 control samples, with significantly lower expression in AD samples. **(E)** The bar chart shows the expression levels of SRP14, EIF3E, and COX7C in AD and control samples, confirming the downregulation of these genes in AD. Statistical significance thresholds: *p < 0.05, **p < 0.01, ***p < 0.001.

To identify key cells, scRNA-seq and cell clustering analysis were performed. Initially, ineligible cells were filtered out, leaving only eligible cells for further analysis (Supplementary Figure S2). A set of 2000 HVGs was identified (Supplementary Figure S3), followed by PCA, which revealed no significant batch effects after data integration (Supplementary Figure S4). The first 30 PCs were selected for downstream analysis (Supplementary Figure S5). QC-passed cells were then assigned to 20 distinct clusters using t-SNE (Figure 4A), and nine cell types were annotated, including CD4^+^ T cells, CD8^+^ T cells, natural killer (NK) cells, B cells, natural killer T (NKT) cells, CD4 NKT cells, CD8 NKT cells, mononuclear macrophages, and megakaryocytes (Figure 4B).

Among the cell types, SRP14 (*P* = 3.0532e-22), EIF3E (*P* = 1.5990e-26), and COX7C (*P* = 3.5062e-03) exhibited consistently elevated expression across multiple annotated cell populations, particularly in CD4^+^ T cells. As a result, SRP14, EIF3E, and COX7C were selected as biomarkers, and CD4^+^ T cells were considered key cells for further investigation (Figure 4C). Additionally, in CD4^+^ T cells, transcript abundance of SRP14, EIF3E, and COX7C was significantly reduced in AD samples (Figure 4D), aligning with expression patterns observed in transcriptome datasets. RT-qPCR further confirmed the reduced expression of SRP14 (*P* = 0.0018), EIF3E (*P* = 0.0270), and COX7C (*P* = 0.0430) in AD samples (Figure 4E). The presence of relatively large error bars (representing standard deviation/standard error) was primarily attributed to interindividual heterogeneity within the clinical AD samples. Specifically, two key factors contributed to this variability: the broad age range of 65–88 years, leading to inherent physiological variability, and variations in baseline health status among the samples, with some patients with AD also diagnosed with hypertension or type 2 diabetes. Age-related physiological decline, compounded by oxidative stress from these comorbidities, likely exacerbated fluctuations in the expression of EIF3E, SRP14, and COX7C(15). Despite these variable error bars, the core trends remained consistent, and the observed interindividual heterogeneity objectively reflected the real-world characteristics of clinical AD samples, providing a foundation for subsequent stratified validation studies based on disease stages.

### 3.5 CD4^+^ T cells contributed to AD pathogenesis

**FIGURE 5:**
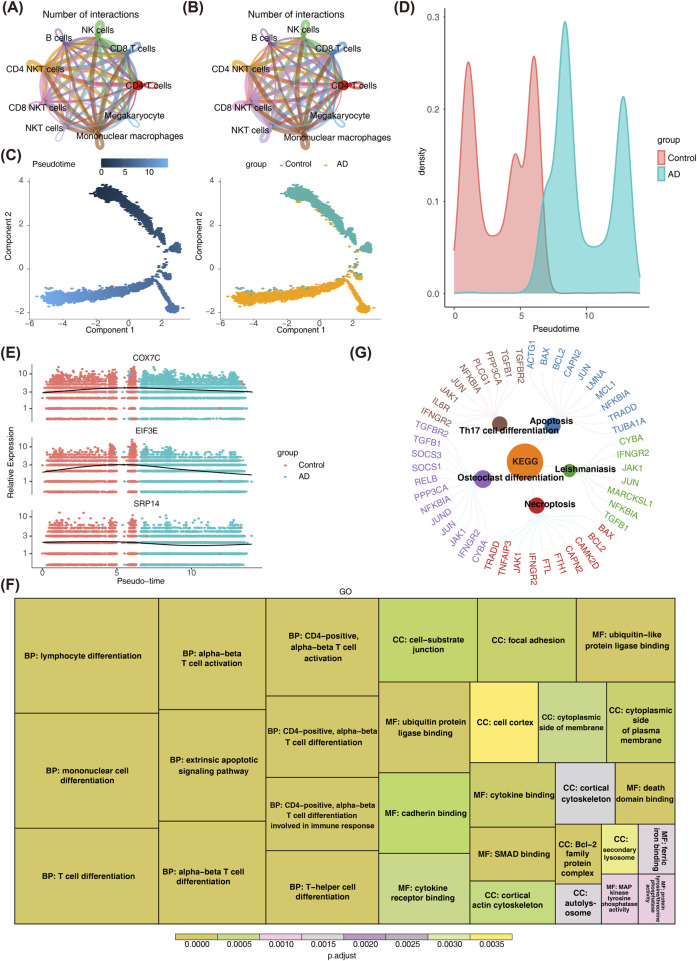
Pseudo-temporal Analysis of CD4 T Cells. **(A,B)** Panel A shows the network diagram of cell interactions in control samples, highlighting significant interactions between CD4 T cells and other cells. Panel B illustrates reduced interactions in AD samples, suggesting potential disruption in cell communication. **(C,D)** Panels C and D display the differentiation stages of CD4 T cells, with cells in the control group predominantly in earlier stages (lighter blue) and AD group cells in later stages. **(E)** Panel E shows the expression levels of key genes during differentiation, which initially increase and then decrease, indicating a dysregulated differentiation process in AD. **(F)** GO enrichment analysis of the top 100 genes. The bar plot reveals significant enrichment in terms such as “mononuclear cell differentiation” and “α-β T cell differentiation.” **(G)** KEGG pathway enrichment analysis of the top 100 genes. The bar plot highlights significant enrichment in pathways like “Th17 cell differentiation” and “apoptosis.”

To investigate the role of CD4^+^ T cells in the pathogenesis of AD, cell communication analysis, pseudotime analysis, and cell enrichment pathway analysis were performed. Cell-to-cell communication analysis in the GSE181279 dataset revealed significant interactions between the key CD4^+^ T cells and other annotated cell types in both AD and control samples (Figures 5A,B; Supplementary Figures S6, S7). Notably, interactions between CD4^+^ T cells and B cells, as well as NKT cells, were more pronounced in control samples than in patients with AD. In the advanced stages of AD, CD4^+^ T cells undergo enhanced differentiation into Th1/Th17 subsets, CD8^+^ T cells either become exhausted or hyperactivated (Afsar et al., 2023), and NK cells exhibit impaired functionality. This disruption of their collaborative interaction (e.g., reduced cell-cell communication) (Li et al., 2024) leads to “uncontrolled inflammation and failure of pathological clearance,” ultimately accelerating neuronal death and cognitive decline. These findings align with our results.

Pseudotime analysis of CD4^+^ T cells indicated that cells in the control group predominantly resided in earlier stages of differentiation, while CD4^+^ T cells from patients with AD were primarily in more advanced stages (Figures 5C,D). Prior to this, our integrated PCA plot (Supplementary Figure S4) showed that cells from AD samples and control samples were mixed in the dimensionality-reduced space, with no obvious group clustering observed. This confirms that batch effects have been effectively controlled. The expression levels of COX7C, EIF3E, and SRP14 showed an initial increase followed by a decrease during the differentiation process of CD4^+^ T cells (Figure 5E).

Additionally, the top 100 genes contributing to CD4^+^ T cell differentiation were analyzed (Supplementary Figure S8). GO analysis revealed significant enrichment in terms related to “mononuclear cell differentiation,” “α-β T cell differentiation,” “ubiquitin-like protein ligase binding,” and “cell-substrate junction” (Figure 5F). These results suggest that these genes may influence CD4^+^ T cell dysfunction and neuroinflammation in AD by regulating CD4^+^ T cell maturation, microenvironmental anchoring, and post-translational modifications. KEGG pathway analysis further identified significant enrichment in pathways related to “Apoptosis,” “Th17 cell differentiation,” “Leishmaniasis,” “Necroptosis,” and “Osteoclast differentiation” (Figure 5G). These abnormalities in AD may lead to the skewing of CD4^+^ T cells toward pro-inflammatory subsets, imbalance in cell survival, and the amplification of neuroinflammation, which exacerbates neuronal damage.

### 3.6 Nomogram demonstrates robust predictive performance

**FIGURE 6:**
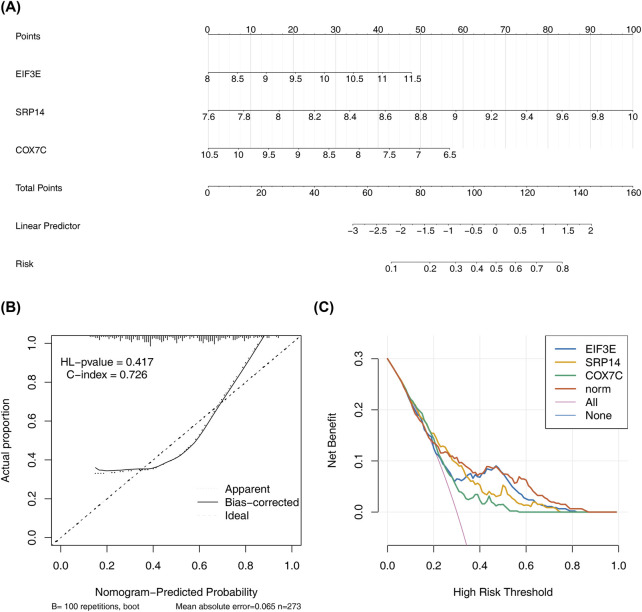
Nomogram Construction and Validation for Disease Risk Prediction. **(A)** The nomogram is based on the expression levels of three key genes. Each gene corresponds to a score on the nomogram,and the higher total score indicates the higher probability of predicting AD. **(B)** The calibration plot demonstrates the concordance of nomogram-derived probabilities with observed event frequencies. **(C)** The DCA curve shows the net benefit of using the nomogram for AD risk prediction compared to using a single factor alone. The curve indicates a higher net benefit from the nomogram.

To assess the predictive potential of the biomarkers, a nomogram was constructed based on the identified biomarkers (Figure 6A). The nomogram demonstrated that higher total scores correlated with an increased risk of AD. The calibration curve showed an HL test *P*-value of 0.417, indicating good agreement between predicted and actual probabilities. The model’s C-index of 0.726 confirmed its effectiveness in distinguishing between the AD and control groups (Figure 6B). Furthermore, the decision curve revealed a higher net benefit from the model compared to using a single factor alone, highlighting its diagnostic utility (Figure 6C). These results highlight the strong predictive capacity of the nomogram.

### 3.7 The exploration of functions of biomarkers

**FIGURE 7:**
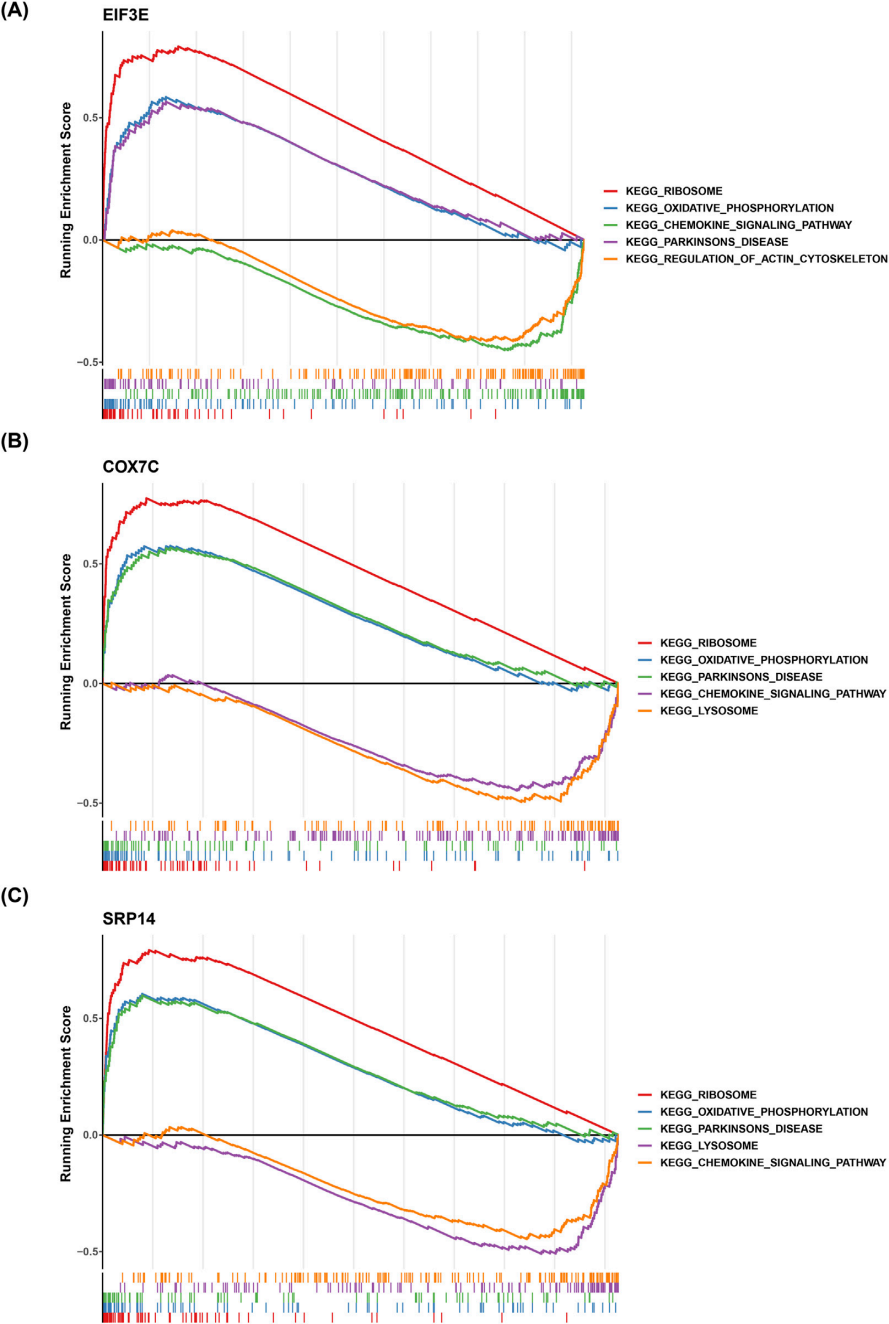
Functional Analysis of Key Genes. **(A-C)** Panels A, B, and C show the enrichment of key genes in the “ribosome,” “oxidative phosphorylation,” and “Parkinson’s disease” pathways, respectively. The results indicate significant co-enrichment of key genes in these pathways.

GSEA was performed to contextualize the biomarkers within well-established biological pathways, avoiding isolated gene analysis. The GSEA results showed significant co-enrichment of EIF3E, COX7C, and SRP14 in pathways such as “ribosome,” “oxidative phosphorylation,” “Parkinson’s disease,” and “chemokine signaling pathway” (Figures 7A–C). This suggests that these three biomarkers may collectively contribute to the pathological progression of AD by regulating protein synthesis, mitochondrial energy metabolism, shared mechanisms in neurodegenerative diseases, and intracerebral immune inflammation.

## 4 Discussion

AD, a neurodegenerative disorder, is characterized by pathological changes that disrupt neuronal integrity and synaptic function, ultimately resulting in cognitive decline (Scheltens et al., 2021). Lysosomal dysfunction plays a pivotal role in the accumulation of AD-associated pathological aggregates and contributes to neurodegeneration (Portin and Wilkins, 2017; Bi et al., 2018). Specifically, the dysregulation of lysosomal ion channels impairs the lysosomal degradative capacity, accelerating AD progression (Riederer et al., 2023). Therefore, lysosomal ion channels serve as crucial molecular nodes, linking proteostasis disruption to cognitive decline in AD. Using the GEO database, this study applied comprehensive bioinformatics analyses to identify three key genes—SRP14, EIF3E, and COX7C. Enrichment analysis, immune infiltration profiling, and regulatory network analysis provide new insights into AD pathogenesis and potential diagnostic and therapeutic avenues.

Genes, as fundamental units of hereditary information, control cellular processes by encoding proteins or regulatory RNAs, with their spatiotemporal expression tightly regulated by epigenetic modifications, transcription factors, and post-transcriptional mechanisms (Portin and Wilkins, 2017). In AD, dysregulation of specific genes has been implicated in key pathological pathways. For example, COX7C, a nuclear-encoded subunit of the mitochondrial cytochrome c oxidase complex, is consistently downregulated in AD brains and peripheral tissues (Bi et al., 2018; Katsumata et al., 2022). This downregulation correlates with impaired mitochondrial respiration, elevated oxidative stress, and reduced Aβ clearance, as indicated by diminished ATP synthesis and aberrant ROS accumulation in neuronal models (Figure 3A). Mechanistically, genetic variants near the COX7C locus may disrupt its transcriptional regulation, further exacerbating bioenergetic deficits in AD.

Similarly, SRP14, a component of the signal recognition particle, exhibits dynamic expression patterns in neurodegenerative diseases such as amyotrophic lateral sclerosis (ALS) and frontotemporal dementia (FTD) (Brown et al., 2020). SRP14 regulates TDP-43 proteostasis by modulating its translation and stress granule dynamics, influencing pathological protein aggregation (Faoro and Ataide, 2021; Chen et al., 2017). SRP14 may further compromise lysosomal function in AD by disrupting the secretion of cathepsins—key enzymes involved in Aβ degradation—a mechanism distinct from its role in ALS/FTD. Despite these advances, the roles of SRP14 and EIF3E in AD-associated lysosomal dysfunction had not been explored until this study.

Single-cell analysis identified CD4^+^ T cells as key mediators of LICRG dysregulation in AD, displaying altered differentiation states and impaired cell-cell communication. This contrasts with Parkinson’s disease (PD), where TMEM175 (another lysosomal ion channel) is upregulated to mitigate mitochondrial stress, highlighting disease-specific regulatory differences (Wie et al., 2021). Moreover, the nomogram model incorporating these biomarkers demonstrated robust predictive accuracy (AUC = 0.726, 95% CI: 0.682–0.769), validated through calibration curves and decision curve analysis (DCA). These findings reveal novel molecular links between lysosomal channels and immune dysregulation in AD, offering a translatable framework for risk stratification and therapeutic targeting.

Building on the expression profiles of SRP14, EIF3E, and COX7C, this study identifies CD4^+^ T cells as a key cellular subset in AD. Interactions between CD4^+^ T cells and NK cells/CD8+ T cells were significantly reduced compared to controls. Additionally, AD-associated CD4^+^ T cells were predominantly arrested at the late differentiation stage. This phenotypic divergence correlates with the dynamic expression pattern of LICRGs, which peak during intermediate differentiation and decline at terminal stages. While previous studies have implicated CD4^+^ T cells in AD-related neuroinflammation, their mechanistic roles remain unclear due to individual variability and limited longitudinal analyses (Afsar et al., 2023).

Our multi-omics approach—integrating bulk transcriptomics, single-cell resolution, and immune cell mapping—uncovers how LICRG dysregulation in CD4^+^ T cells disrupts intercellular crosstalk, potentially exacerbating Aβ deposition through impaired immune surveillance. For instance, weakened communication between CD4^+^ T and NK cells may hinder the cytotoxic clearance of Aβ aggregates, while aberrant differentiation of CD4^+^ T cells toward pro-inflammatory Th17 subsets could amplify neurotoxicity. These findings contrast with PD, where CD8^+^ T cells predominate in neuroinflammation, highlighting the distinct immune pathology of AD (Contaldi et al., 2022). The nomogram model incorporating SRP14, EIF3E, and COX7C expression demonstrated strong diagnostic accuracy (AUC: 0.726), offering a clinically actionable tool for early AD detection. Future research should validate these biomarkers in cerebrospinal fluid (CSF) and explore therapeutic strategies to modulate CD4^+^ T-cell differentiation, such as small molecules targeting LICRGs to restore lysosomal-immune homeostasis.

GSEA revealed that SRP14, EIF3E, and COX7C were co-enriched in pathways critical to AD pathogenesis, including ribosome biogenesis, oxidative phosphorylation (OXPHOS), PD-related pathways, and chemokine signaling pathways (Figure 6A). These findings highlight the multifaceted mechanisms linking lysosomal ion channel dysfunction to proteostatic collapse, metabolic failure, and neuroinflammation in AD.

Abnormal activation of the ribosome collision stress pathway induces metabolic imbalance and collapses protein homeostasis, accelerating neuronal degeneration and driving Aβ/tau pathological deposition, thereby exacerbating disease progression (Wu et al., 2020). OXPHOS impairment in AD microglia is closely linked to Aβ-induced glycolytic metabolic reprogramming and the H4K12la/PKM2 positive feedback loop, which further exacerbates energy imbalance and promotes pathological protein deposition. Targeting this metabolic axis may restore OXPHOS function and mitigate neurodegenerative changes (Pan et al., 2022). The chemokine pathway (CX3CL1/CX3CR1 and P2X7R signaling axis) plays a critical role in regulating microglial activation states (Suresh et al., 2021). In AD, dysregulated CX3CL1 signaling impairs microglial Aβ phagocytosis while enhancing IL-1β release, amplifying neuroinflammation (Suresh et al., 2021). Conversely, P2X7R antagonism has been shown to restore synaptic integrity in preclinical models (Suresh et al., 2021), suggesting context-dependent roles for chemokine signaling.

Notably, our data revealed striking contrasts between AD and PD, despite shared pathways such as mitochondrial dysfunction. For instance, COX7C and EIF3E, which are downregulated in AD, are upregulated in PD to counteract oxidative stress. Similarly, TMEM175, a lysosomal K+ channel that is neuroprotective in PD through pH modulation, is suppressed in AD, exacerbating lysosomal-mitochondrial miscommunication. These opposing expression patterns (Figure 6C) suggest that neurodegenerative diseases co-opt common pathways through divergent regulatory mechanisms, which is an important consideration for targeted therapies.

Through the integration of multi-omics approaches, including differential expression analysis, WGCNA, and scRNA-seq, this study identified SRP14, EIF3E, and COX7C as core biomarkers linked to lysosomal ion channel dysregulation in AD. GSEA further revealed that these biomarkers were significantly enriched in key neurodegenerative pathways, including ribosome biogenesis, OXPHOS, and PD-associated signaling, providing novel insights for AD clinical diagnosis and therapeutic targeting. The computational predictions were robustly validated by RT-qPCR in an independent cohort. However, limitations exist, including the need for further experimental validation, the potentially limiting sample size in scRNA-seq for detecting rare immune subsets, and the need for deeper exploration of the precise mechanistic roles of these biomarkers in AD pathogenesis. Future research should focus on elucidating the underlying mechanisms driving AD progression.

## 5 Conclusion

This study identified SRP14, EIF3E, and COX7C as lysosomal ion channel-related biomarkers significantly associated with AD pathogenesis, with CD4^+^ T cells serving as critical mediators of lysosomal-immune dysregulation. Integrated multi-omics analyses revealed dynamic biomarker expression patterns during CD4^+^ T cell differentiation and their enrichment in pathways related to proteostasis collapse (ribosome biogenesis) and metabolic failure (OXPHOS). By linking computational discovery with therapeutic innovation, this work lays the foundation for precision medicine strategies targeting lysosomal-immune homeostasis in AD.

## Data Availability

The transcriptomic datasets (GSE63061, GSE63060, GSE181279) supporting this study are publicly available in the Gene Expression Omnibus (GEO) repository at https://www.ncbi.nlm.nih.gov/geo/. The processed analytical results generated during this study are included in this published article and its Supplementary Information files. The datasets validating the results reported herein are held by the corresponding author and will be shared upon receipt of a reasonable request.
