# Supplementary figures and images for: Integrated transcriptomic and single-cell RNA sequencing identifies lysosomal ion channel genes as potential biomarkers for Alzheimer’s disease
Source: Front Genet. 2025 Oct 8;16:1676565. doi: 10.3389/fgene.2025.1676565 (PMC12542832; doi:10.3389/fgene.2025.1676565)

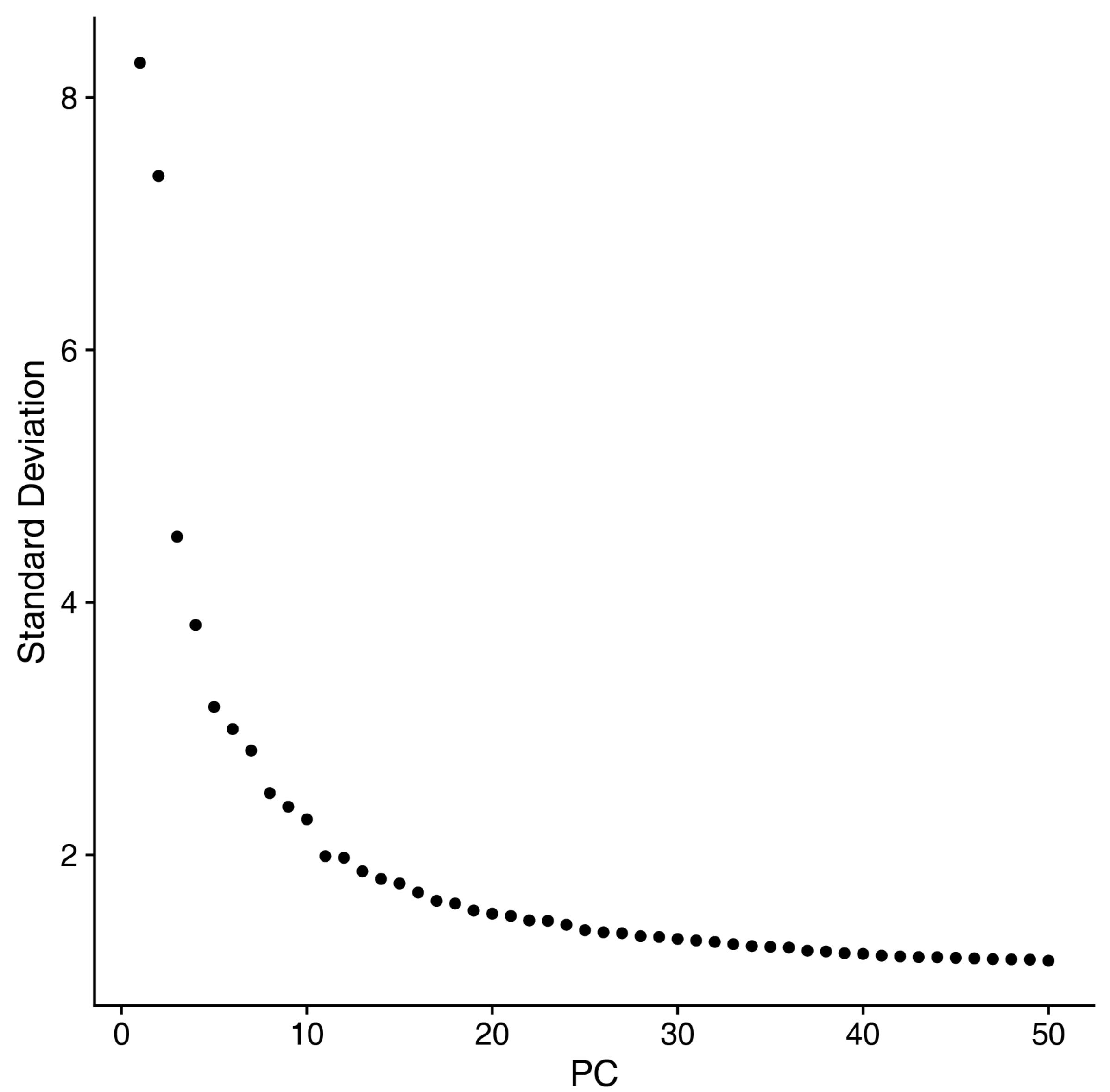

Supplement: Supplementary file 1 [file Image5.pdf]

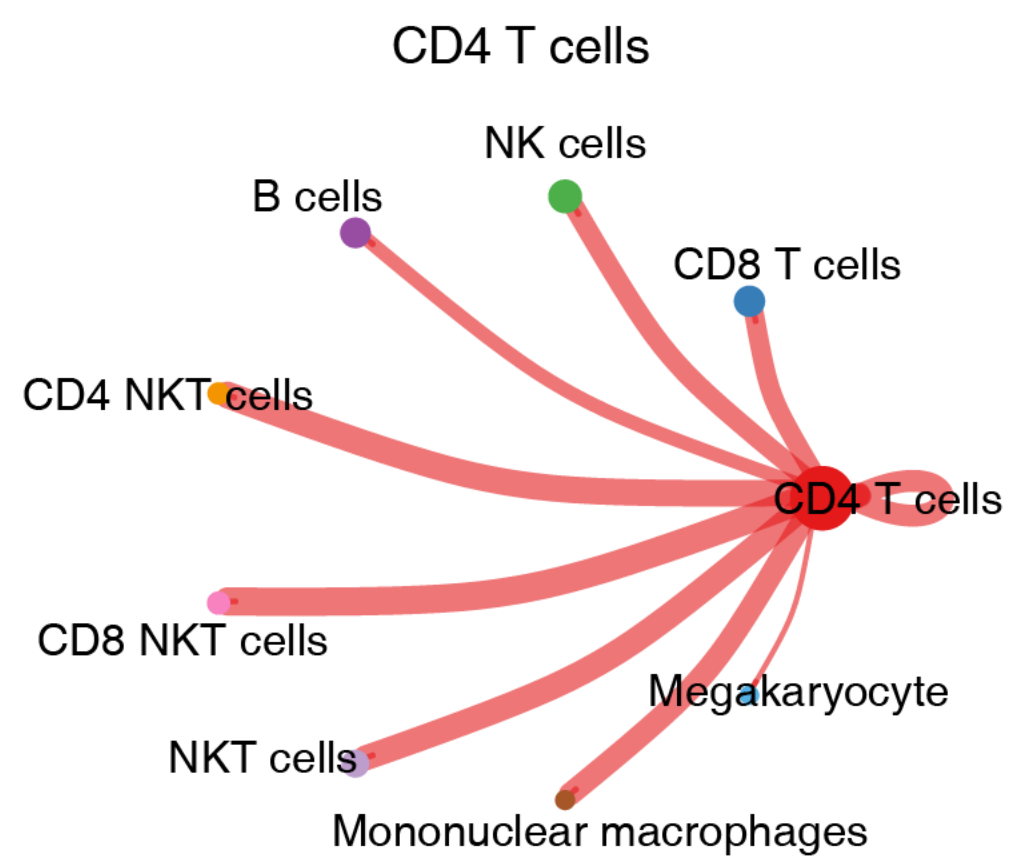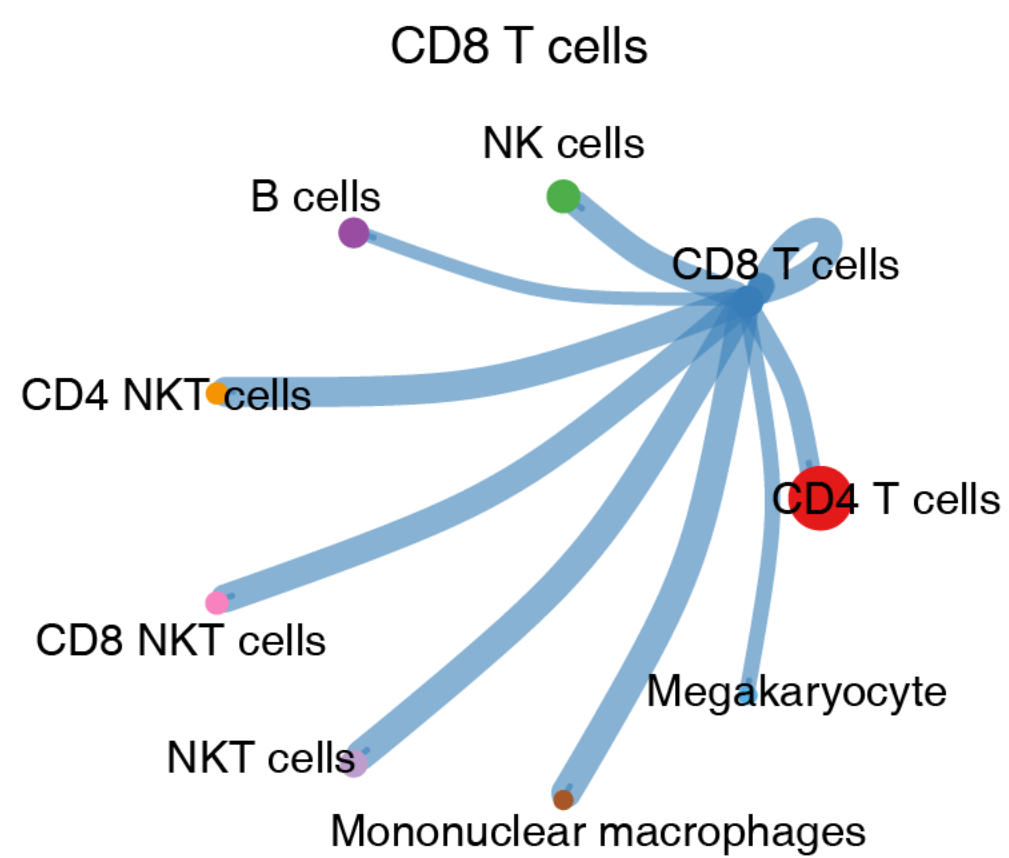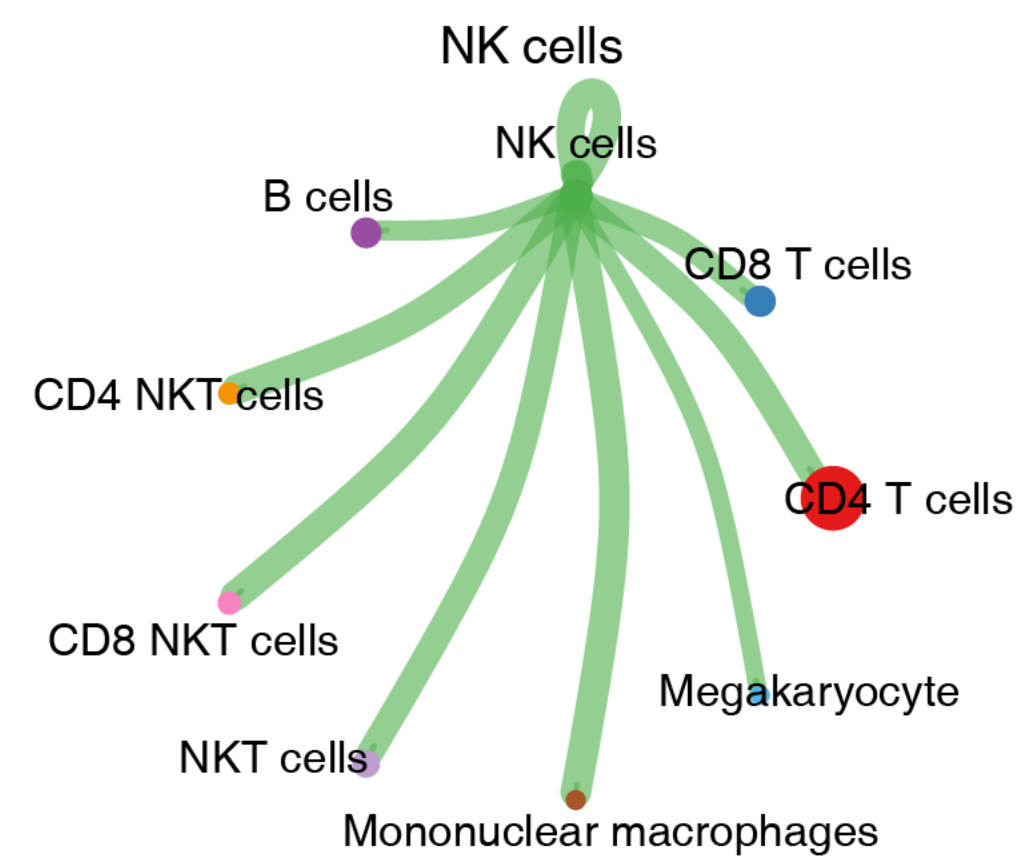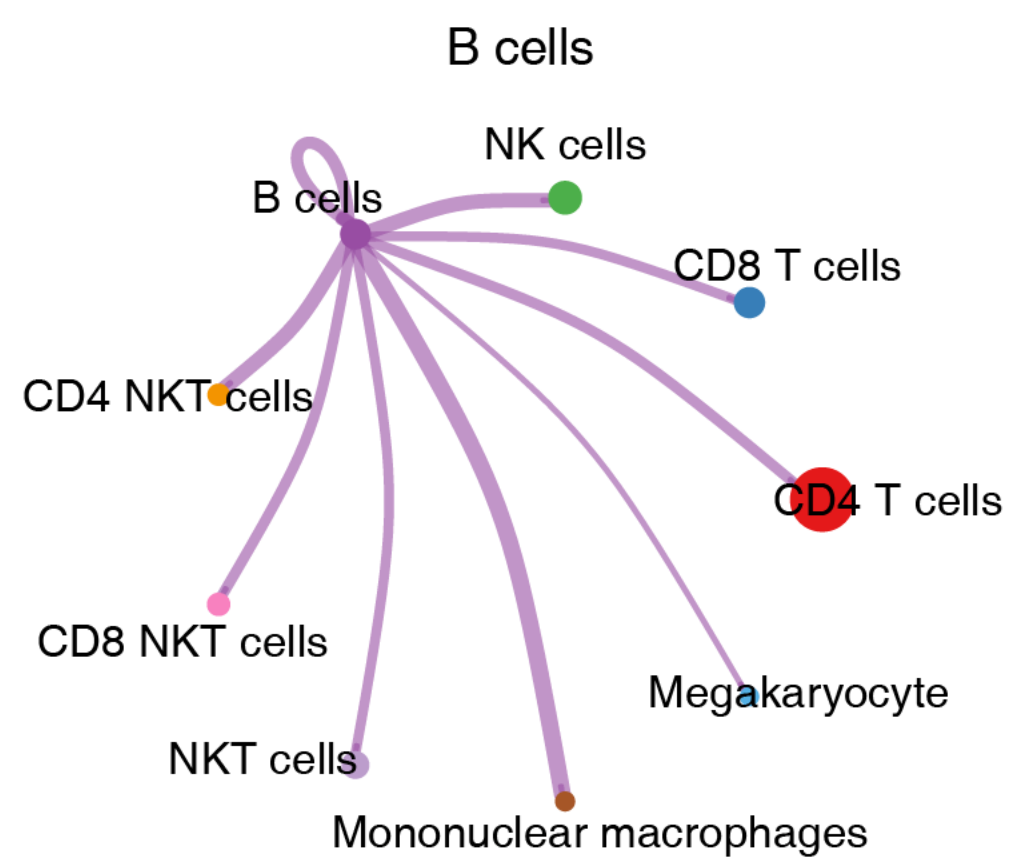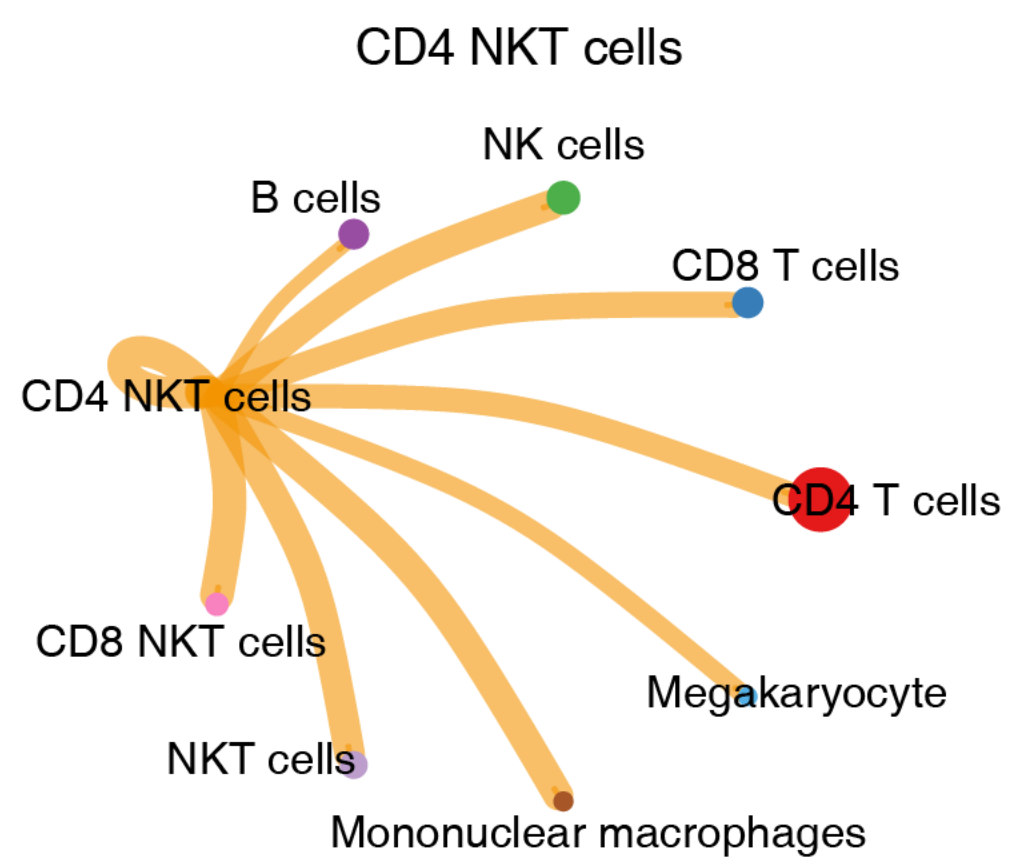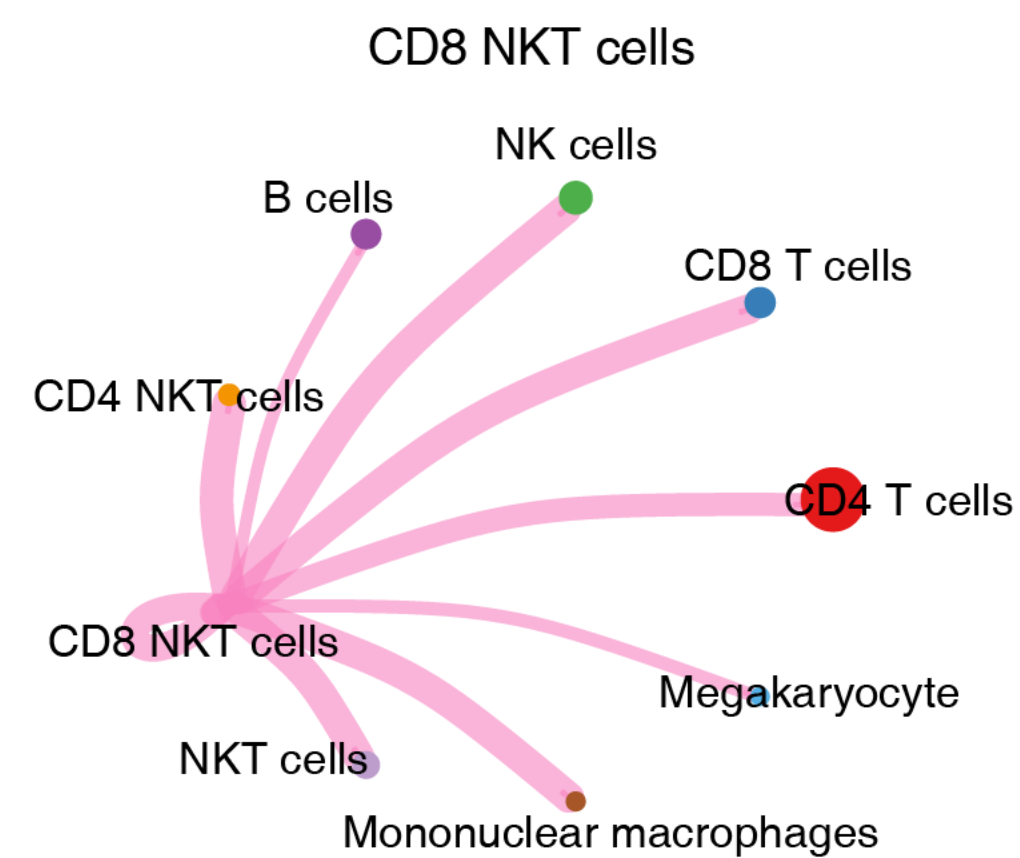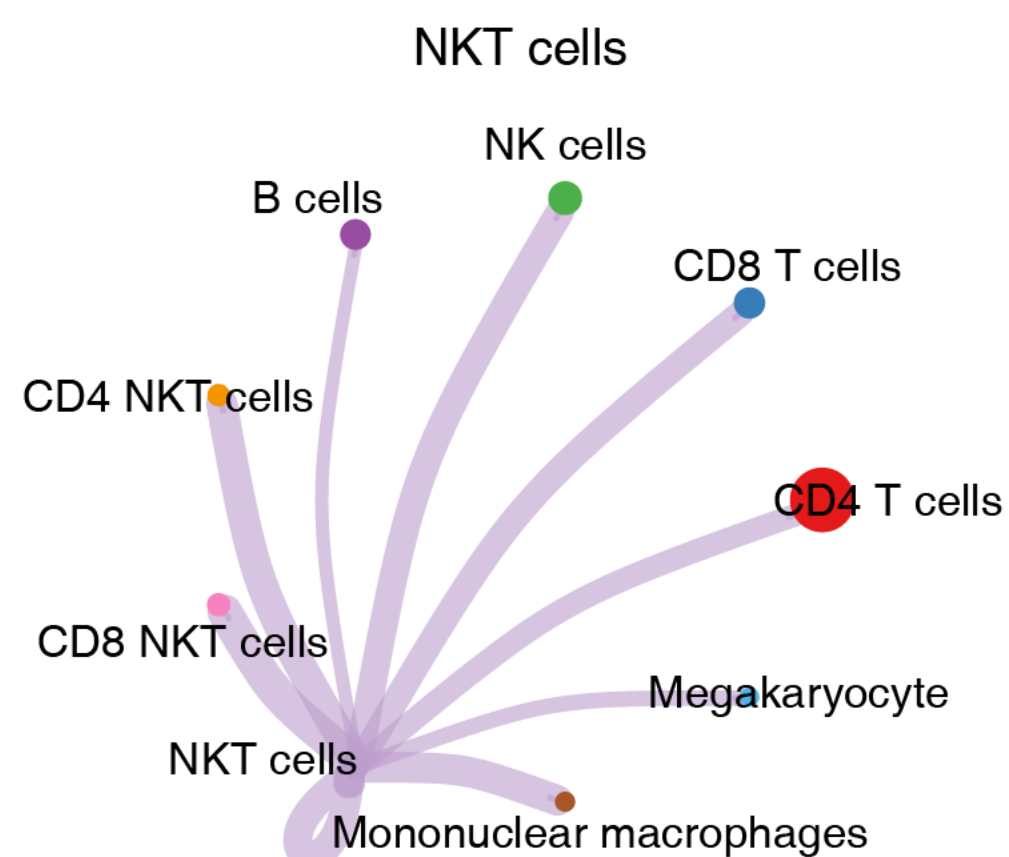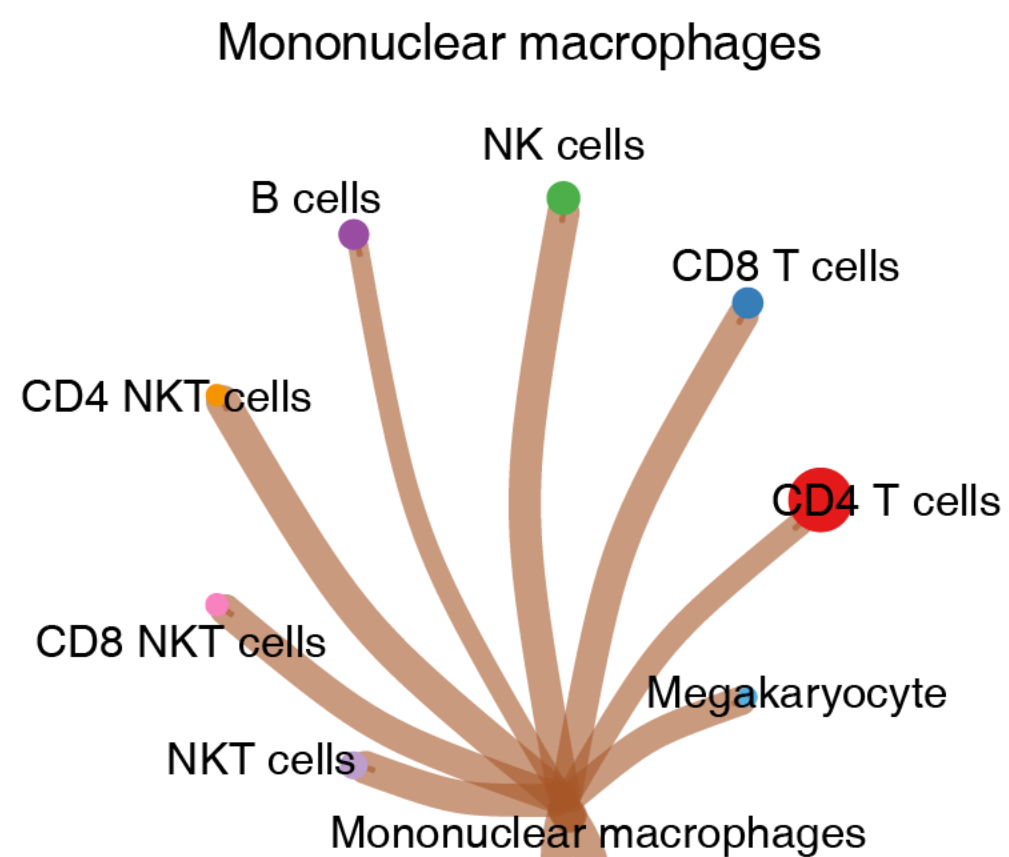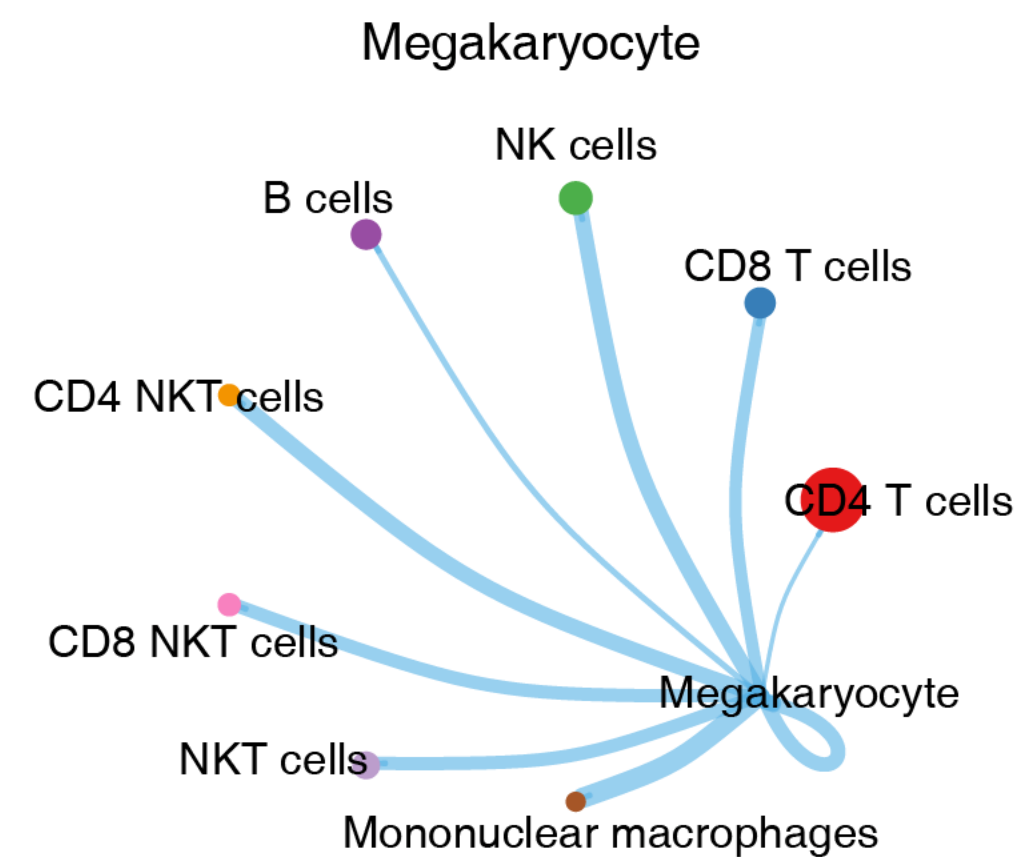

Supplement: Supplementary file 3 [file Image6.pdf]

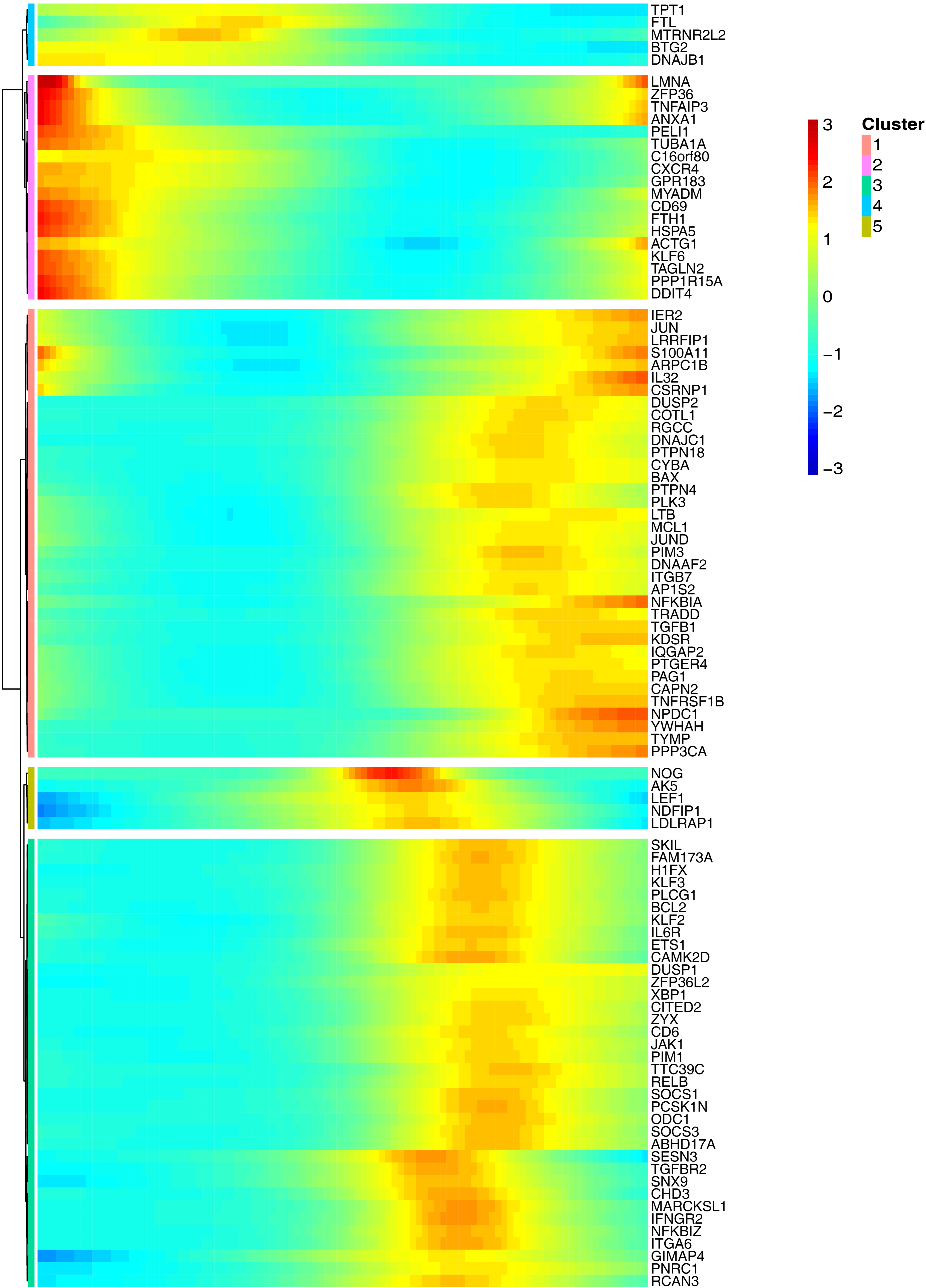

Supplement: Supplementary file 4 [file Image8.pdf]

orig.ident

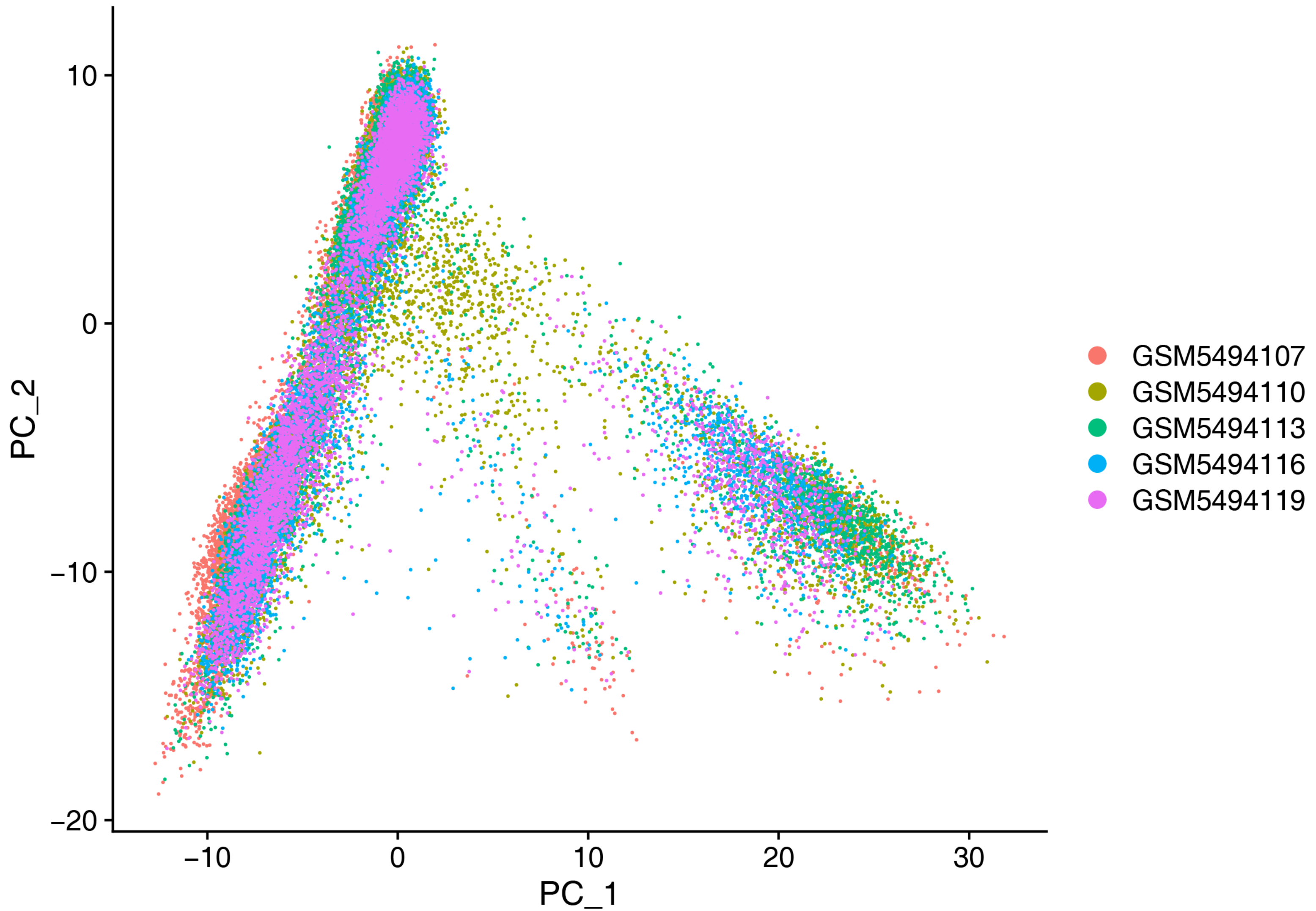

Supplement: Supplementary file 5 [file Image4.pdf]

**nFeature\_RNA**

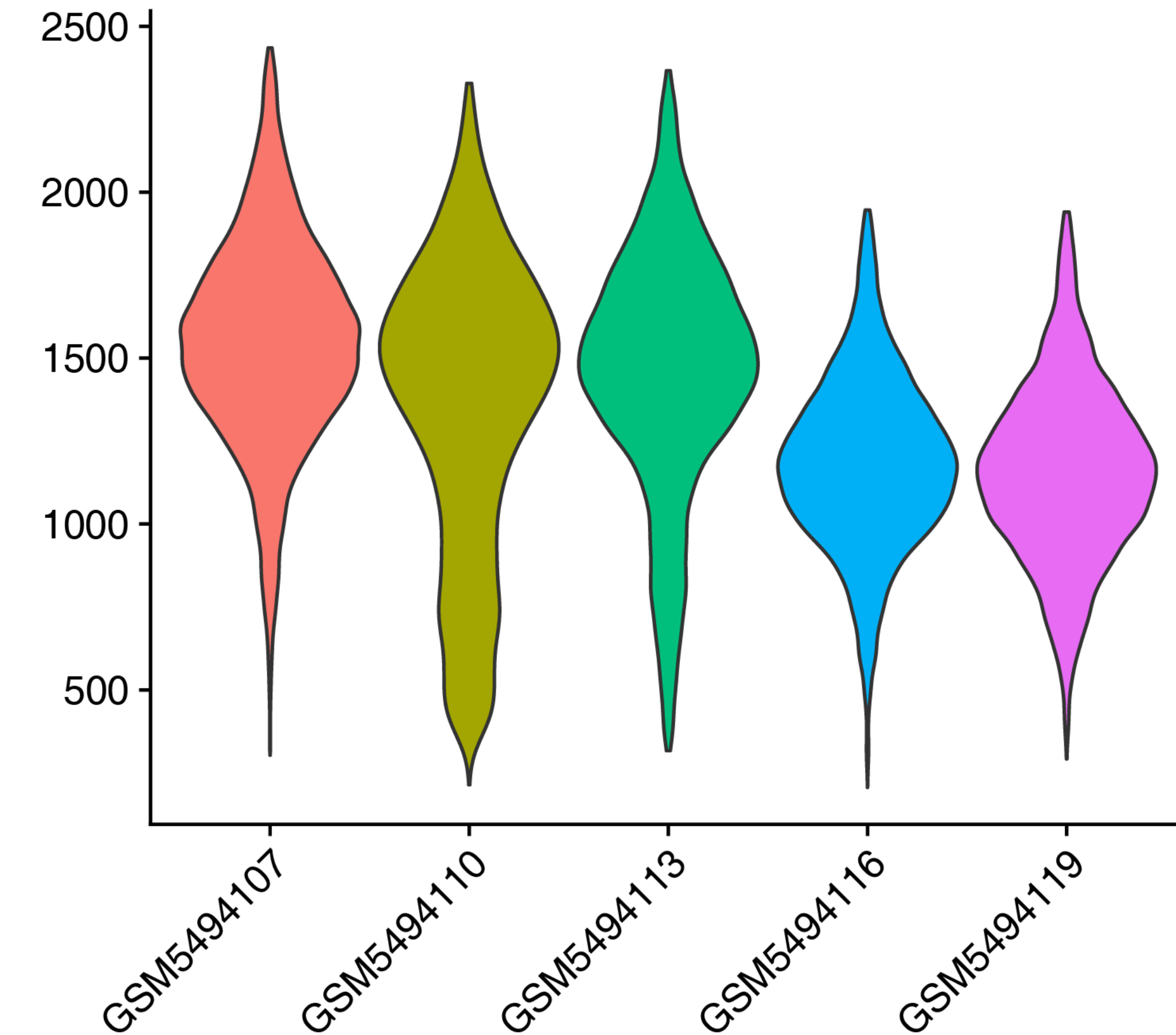

**nCount\_RNA**

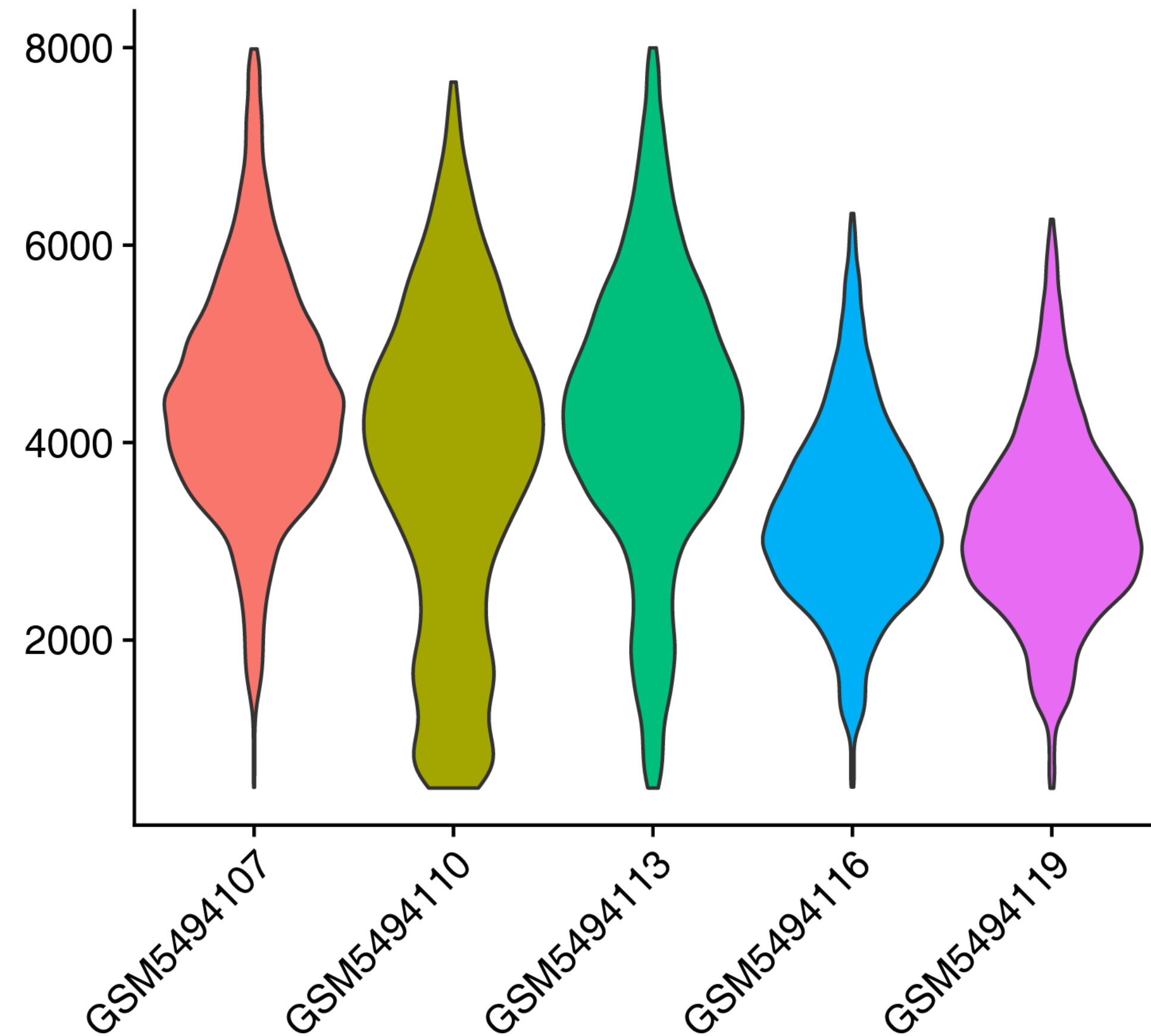

**percent.mt**

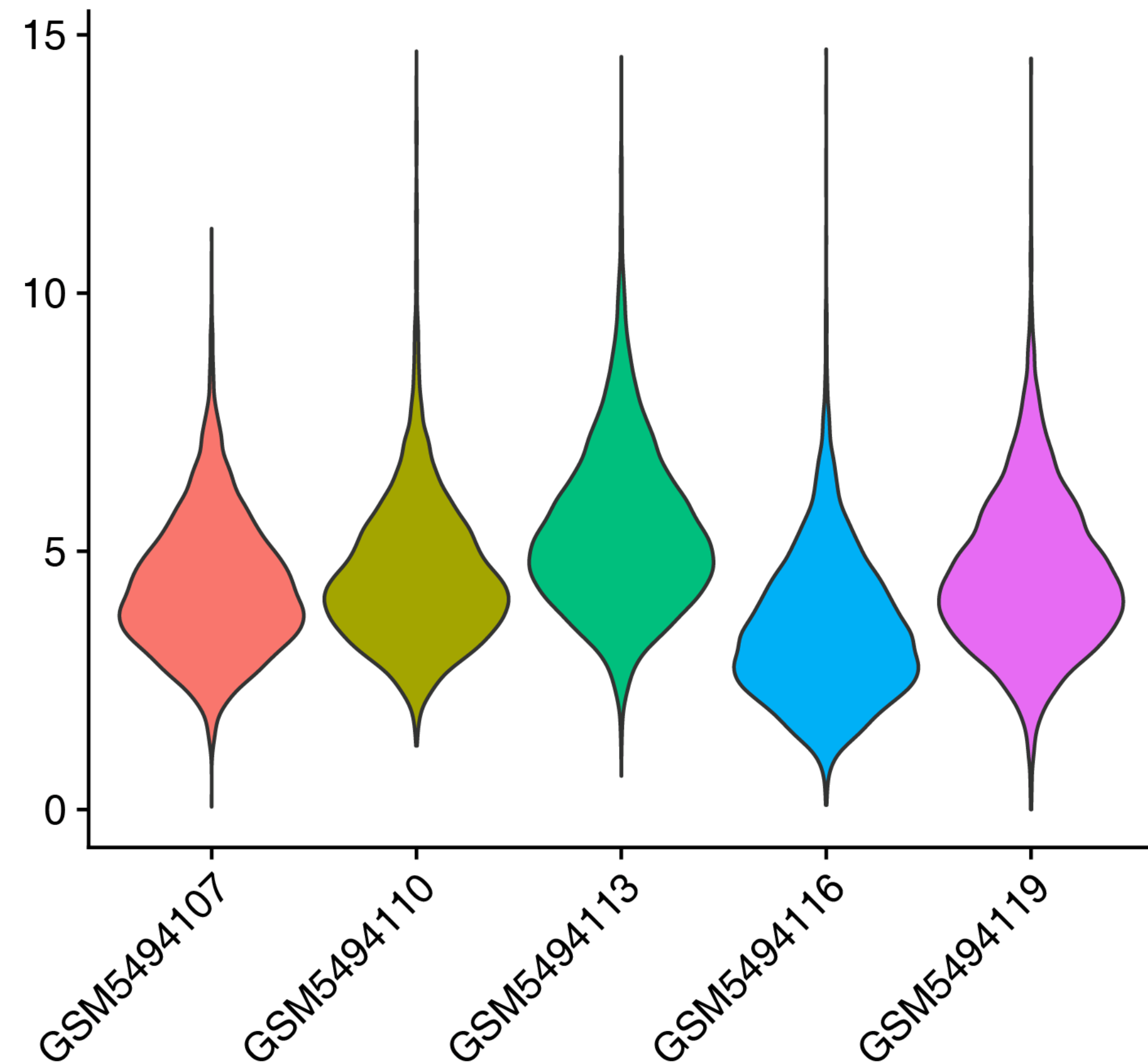

Supplement: Supplementary file 6 [file Image2.pdf]

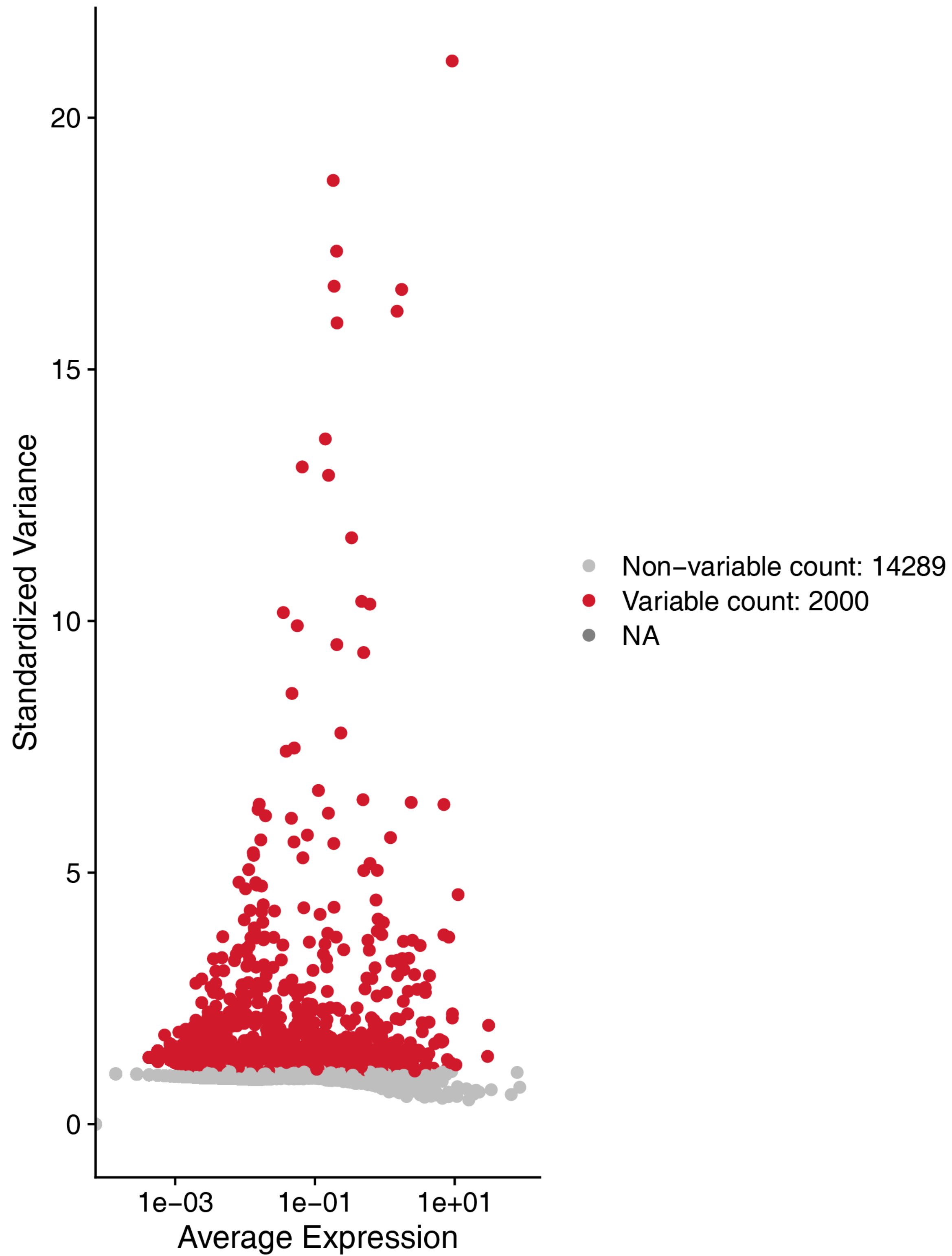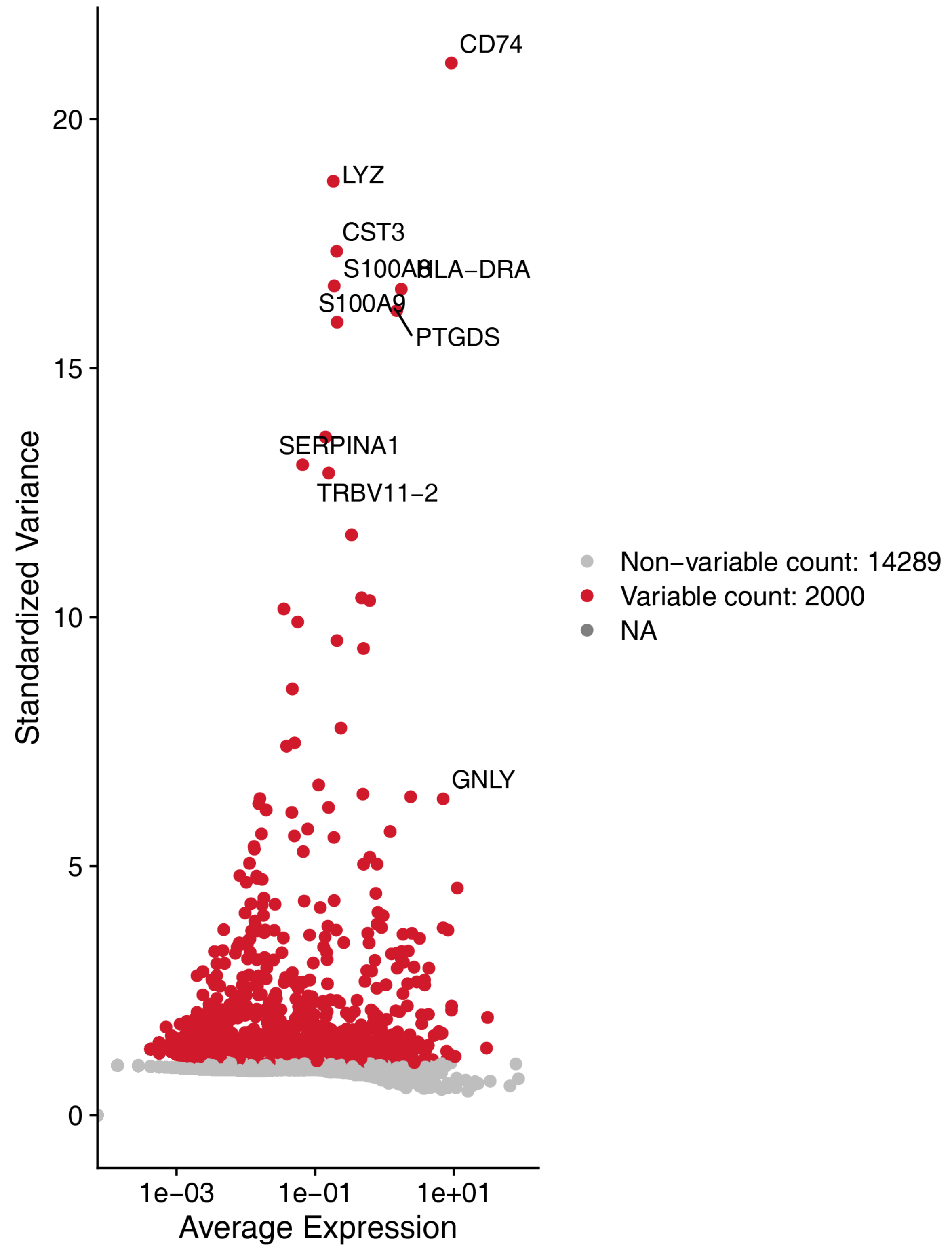

Supplement: Supplementary file 7 [file Image3.pdf]

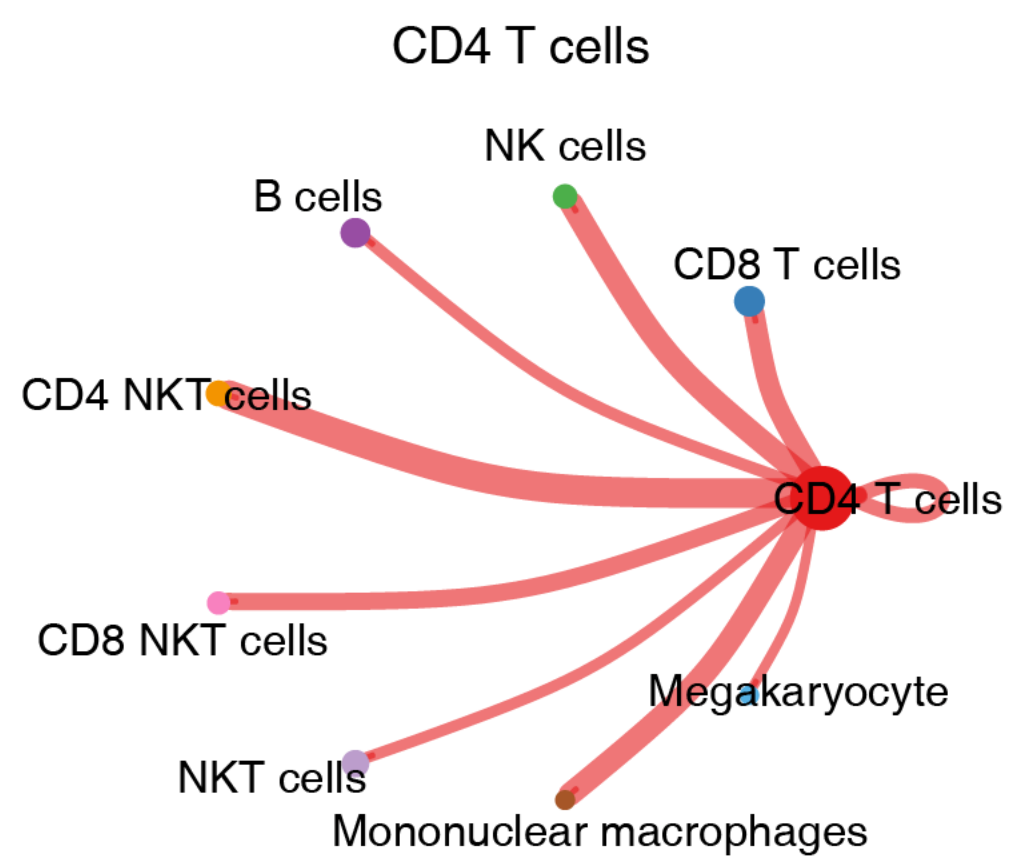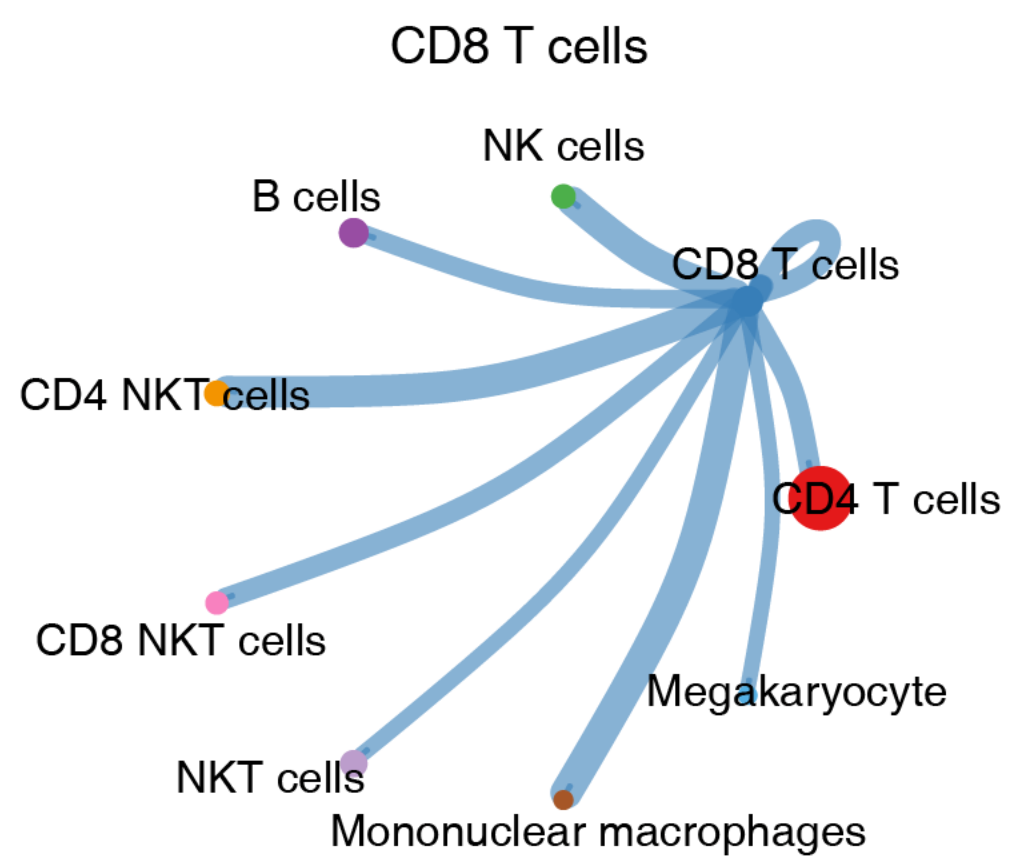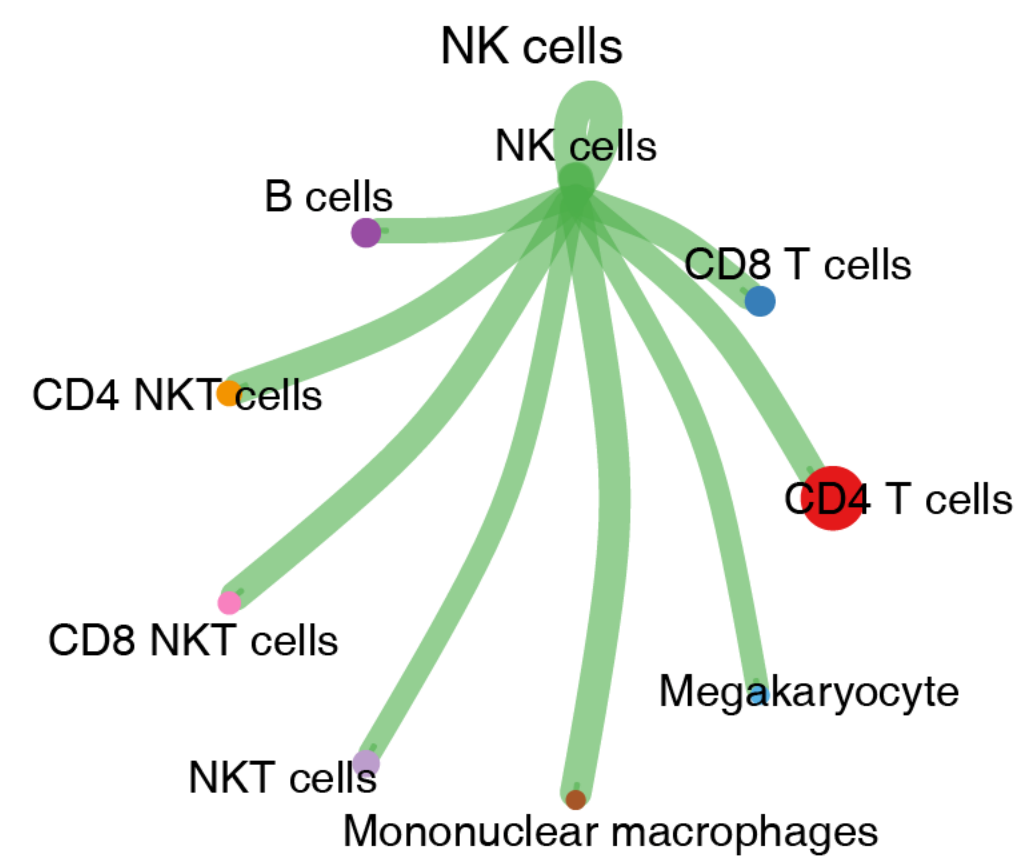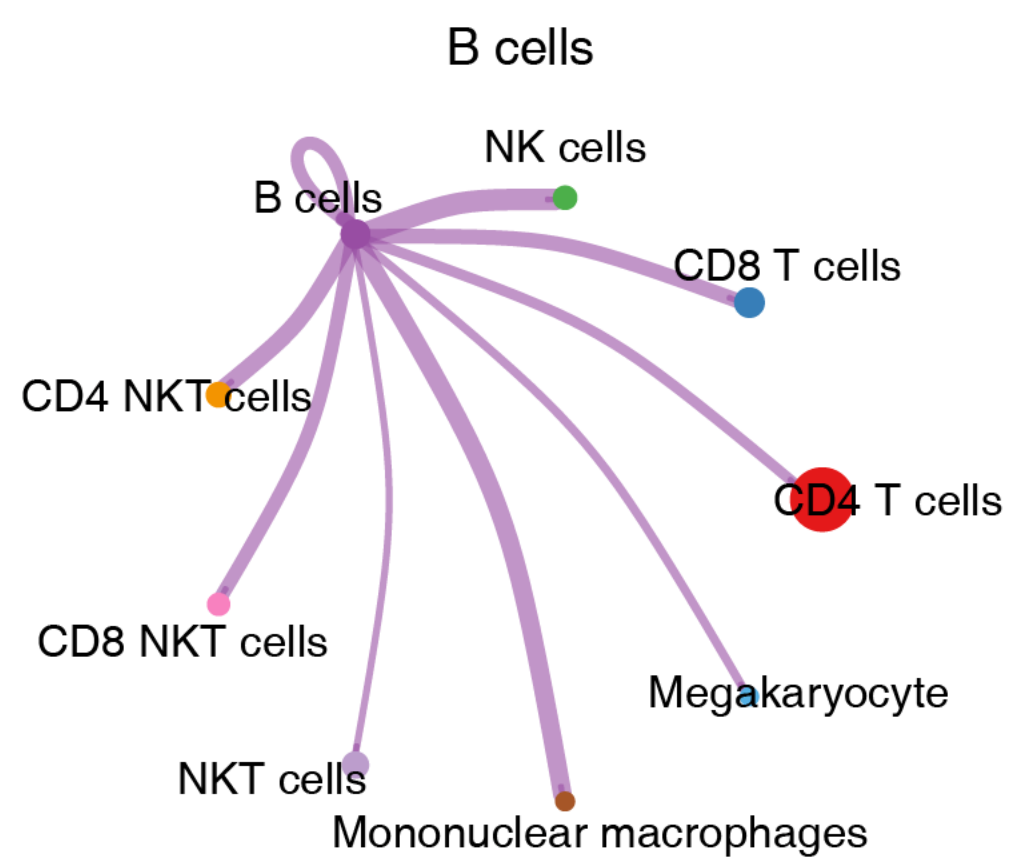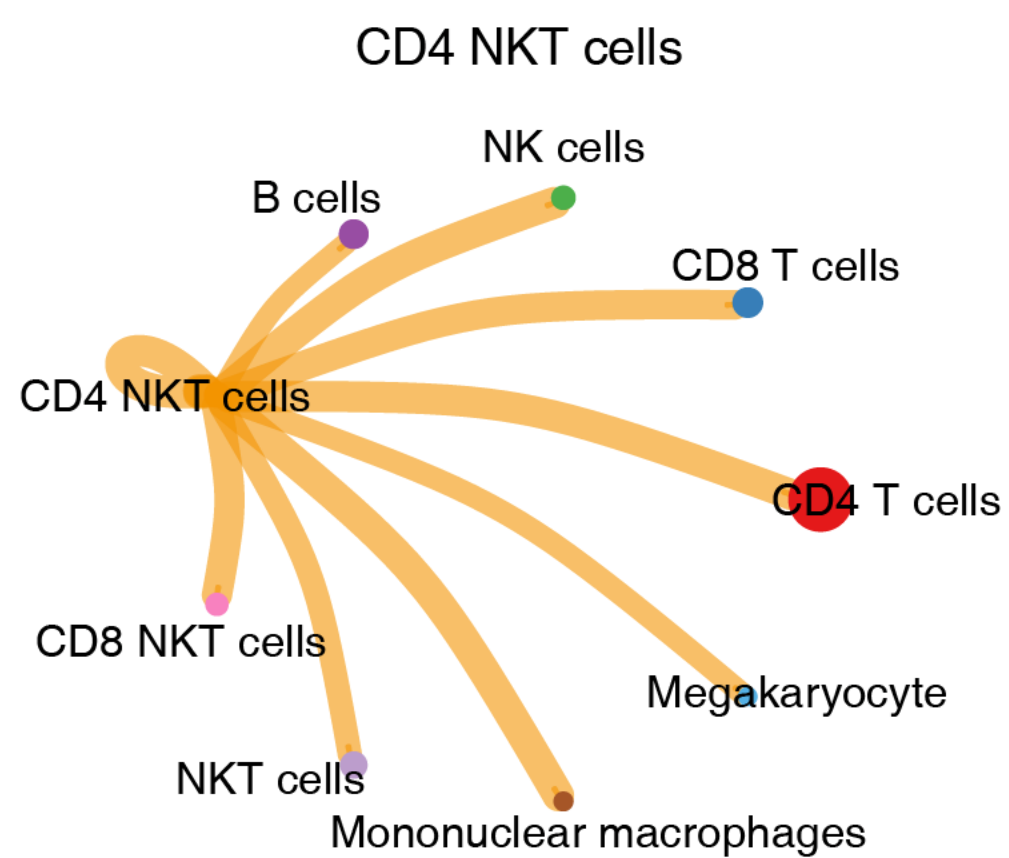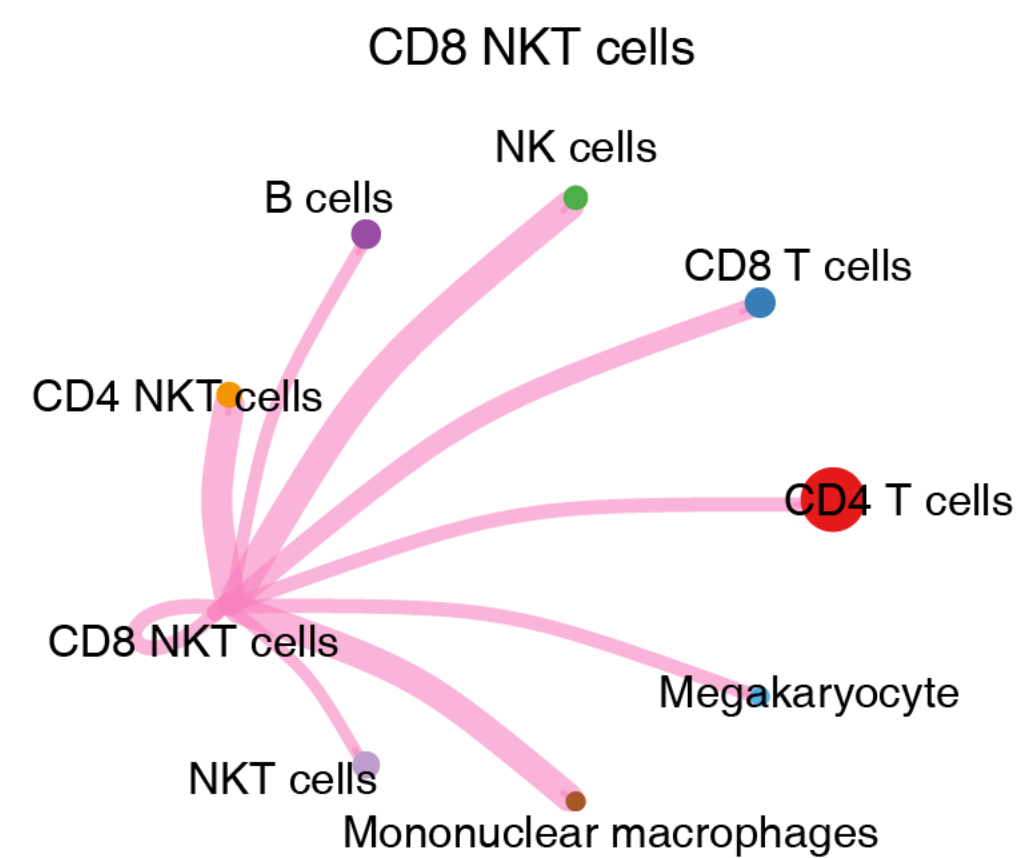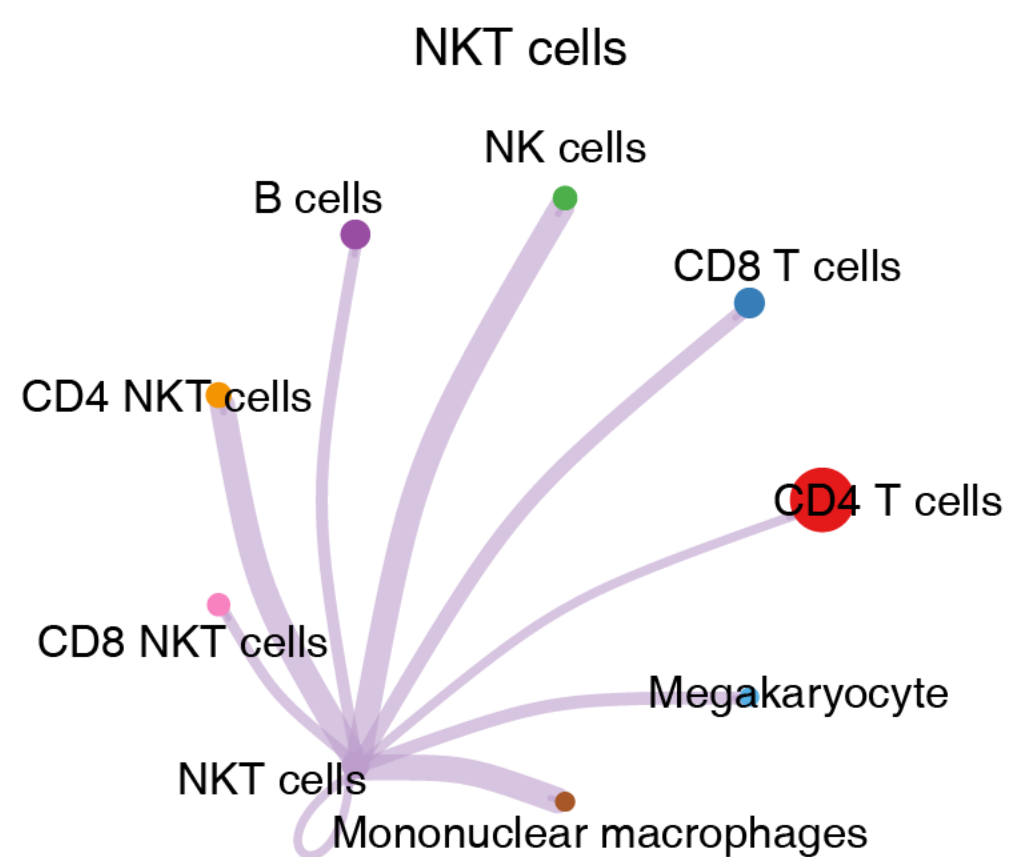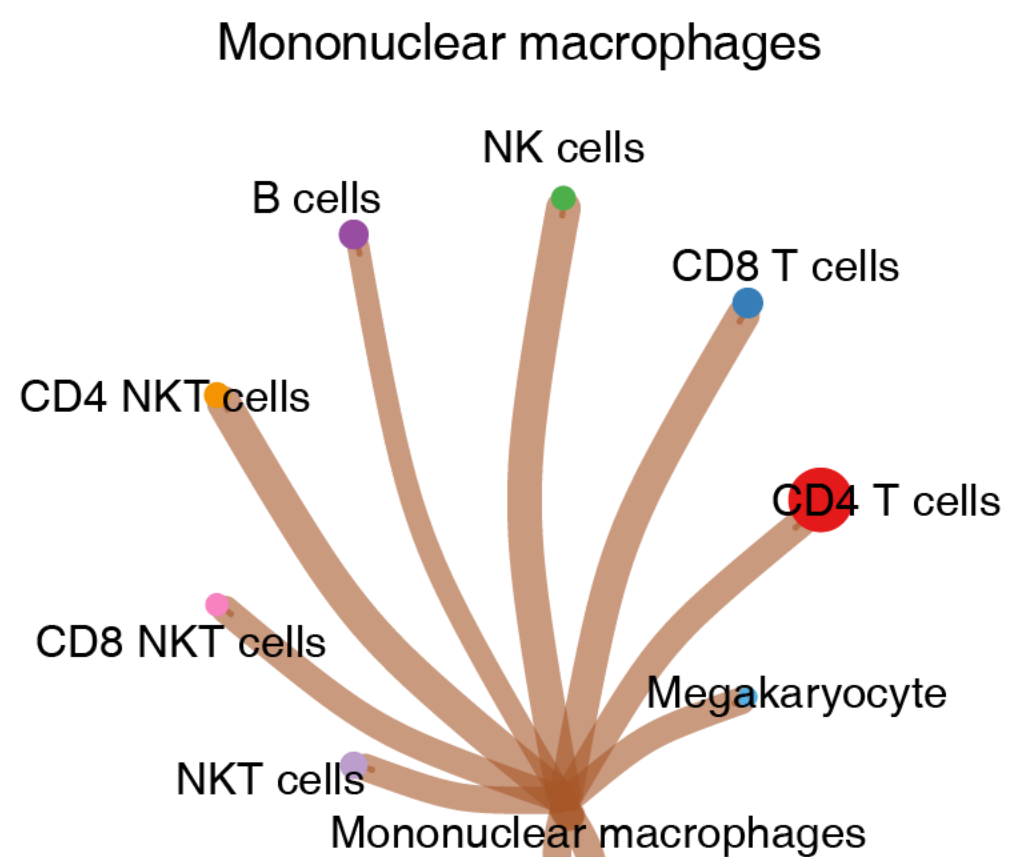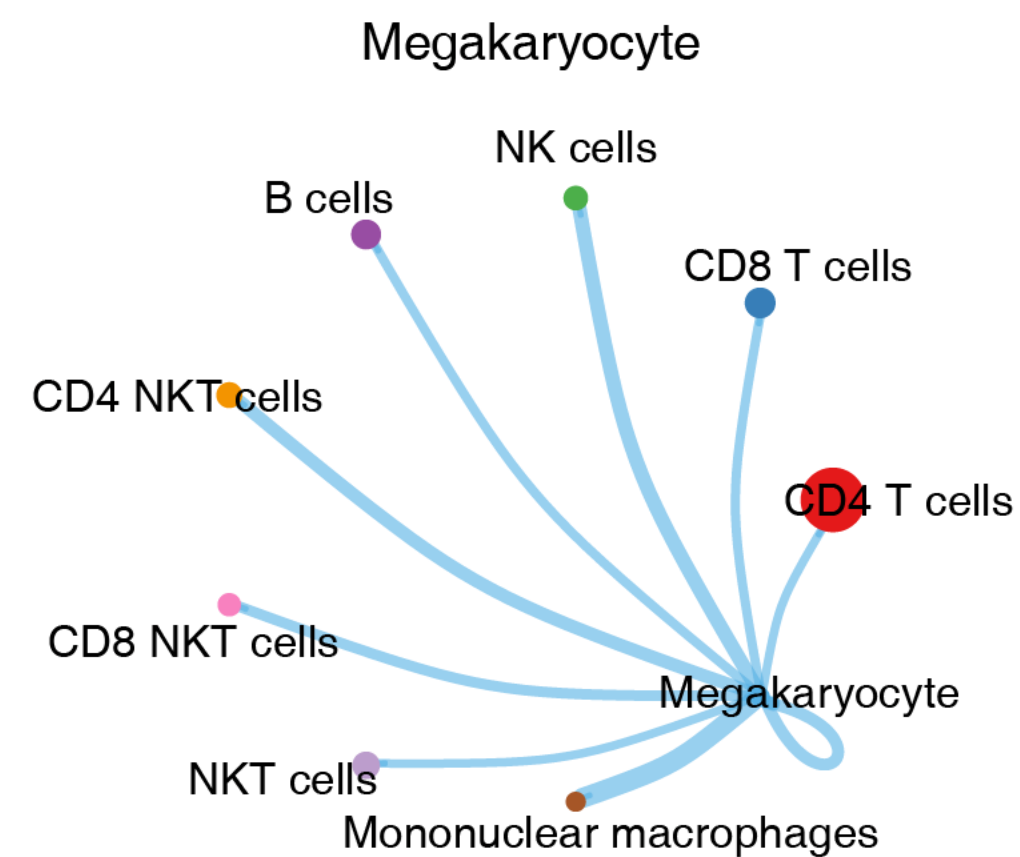

Supplement: Supplementary file 8 [file Image7.pdf]

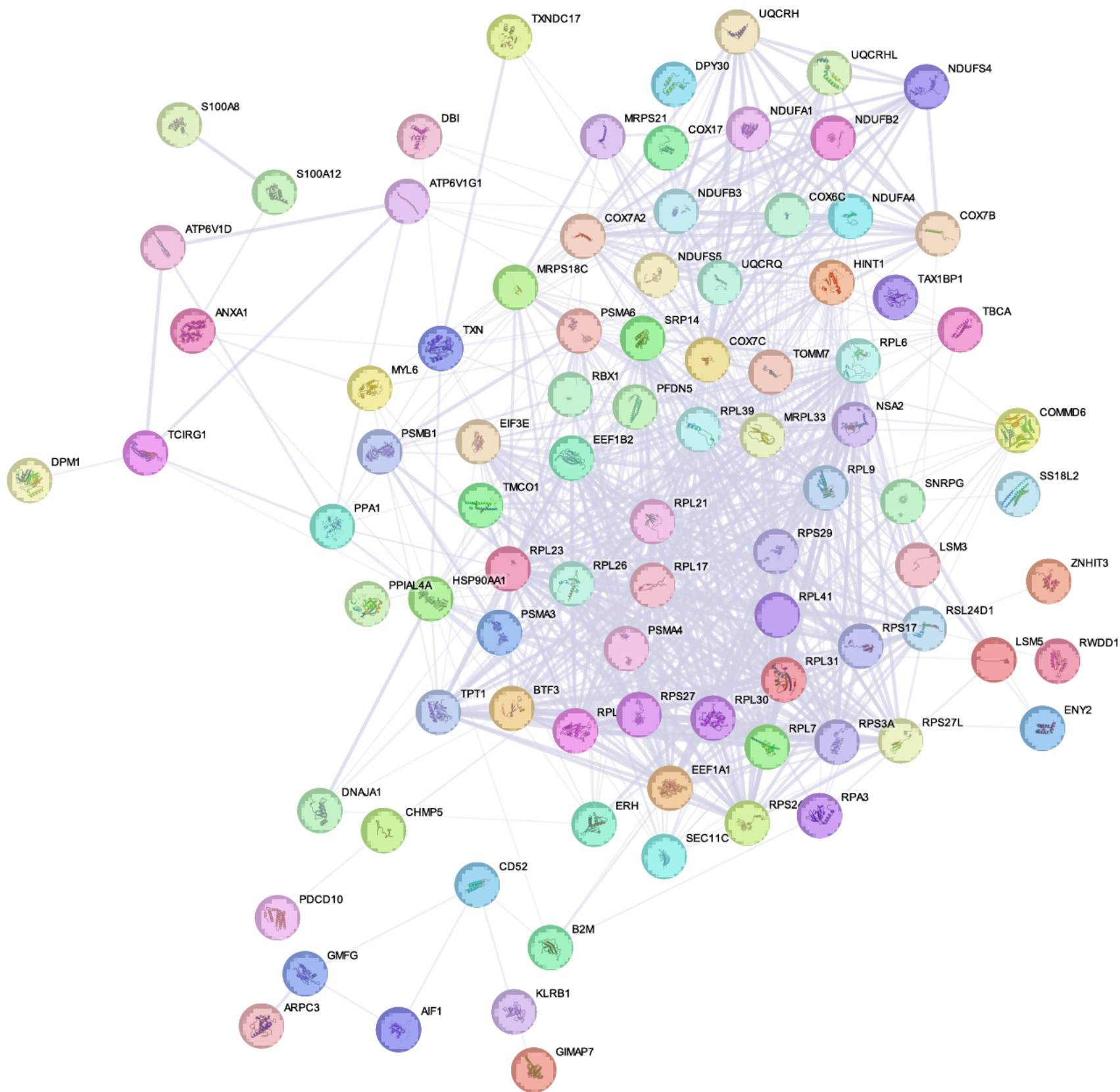

Supplement: Supplementary file 9 [file Image1.pdf]
