# Supplementary material for: Integrated transcriptomic and single-cell RNA sequencing identifies lysosomal ion channel genes as potential biomarkers for Alzheimer’s disease
Source: Front Genet. 2025 Oct 8;16:1676565. doi: 10.3389/fgene.2025.1676565 (PMC12542832; doi:10.3389/fgene.2025.1676565)
Supplement: Supplementary file 2 [file Table1.docx]

| **Primer** | **Sequence** | |
| --- | --- | --- |
| SRP14 F | GACGGAGCTGACCAGACTTT | |
| SRP14 R | TCCTTGGAGCTCACCACAGT | |
| EIF3E F | CGGCATCTAGTCTTTCCGCT |  |
| EIF3E R | CCTGGTTGACTGCATTTGCC |  |
| COX7C F | CAACCTCTGTGGTCCGTAGG |  |
| COX7C R | GGAAGGGTGTAGCAAATGCAG |  |
| Internal reference -GAPDH F | CGAAGGTGGAGTCAACGGATTT |  |
| Internal reference -GAPDH R | ATGGGTGGAATCATATTGGAAC |  |

**The sequences of all primers for RT-qPCR**
